# Ruling In and Ruling Out Sepsis Using Likelihood Ratios of a Host Response Assay

**DOI:** 10.3390/diagnostics16152450

**Published:** 2026-08-03

**Authors:** Krupa Arun Navalkar, Prashant Wani, Roy F. Davis, Silvia Cermelli, Maximilian Dietrich, Maik von der Forst, Sören L. Becker, Sophia Benthien, Elisa Baumann, Carsten Zeiner, Philipp M. Lepper, José Garnacho-Montero, María Luisa Cantón-Bulnes, Adela Fernández-Galilea, Jose Luis García-Garmendia, Ángel Estella, Russell R. Miller, Marcus J. Schultz, Richard Rothman, John Burke, Gourang Patel, Jorge Parada, Thomas D. Yager, Richard B. Brandon

**Affiliations:** 1 Immunexpress Inc., Seattle, WA 98109, USA; prashant.w@immunexpress.com (P.W.); roy.d@immunexpress.com (R.F.D.); silvia.c@immunexpress.com (S.C.); richard.b@immunexpress.com (R.B.B.); 2 Department of Anesthesiology, Medical Faculty Heidelberg, Heidelberg University, Im Neuenheimer Feld 420, 69120 Heidelberg, Germany; maximilian.dietrich@med.uni-heidelberg.de (M.D.); maik.forst@med.uni-heidelberg.de (M.v.d.F.); 3 Institute of Medical Microbiology and Hygiene, Saarland University, 66421 Homburg, Germany; soeren.becker@uks.eu (S.L.B.); sophia.benthien@lab-kl.de (S.B.); elba00007@stud.uni-saarland.de (E.B.); 4 Department of Internal Medicine V, Pneumology and Intensive Care Medicine, Saarland University, 66421 Homburg, Germany; carsten.zeiner@uks.eu; 5 Department of Internal Medicine, Pneumology and Intensive Care Medicine, University Hospital OWL Campus Bethel, University of Bielefeld, 33617 Bielefeld, Germany; philipp.lepper@evkb.de; 6 Intensive Care Unit, Hospital Universitario Virgen del Rocío, 41013 Sevilla, Spain; jgarnachom@gmail.com (J.G.-M.); adelafer_90@hotmail.com (A.F.-G.); 7 Intensive Care Unit, Hospital Universitario Virgen Macarena, 41009 Sevilla, Spain; luisabulnes@hotmail.com; 8 Intensive Care Unit, Hospital San Juan de Dios del Aljarafe, 41930 Sevilla, Spain; joseluis.garciagarmendia@sjd.es; 9 Intensive Care Unit, Hospital Universitario de Jerez de la Frontera, 11407 Cádiz, Spain; angel.estella@gm.uca.es; 10 Instituto de Investigación e Innovación Biomédica de Cádiz (INiBICA), Department of Medicine and Surgery, University of Cádiz, 11003 Cádiz, Spain; 11 Department of Critical Care Medicine, FirstHealth of the Carolinas, Pinehurst, NC 28374, USA; rmiller@firsthealth.org; 12 Department of Anaesthesia, General Intensive Care and Pain Management, Division of Cardiothoracic and Vascular Anaesthesia & Critical Care Medicine, Medical University Wien, 1090 Vienna, Austria; marcus.j.schultz@gmail.com; 13 Department of Anaesthesiology, Rescue and Pain Medicine, Cantonal Hospital St. Gallen, HOCH Health Ostschweiz, 9007 St. Gallen, Switzerland; 14 Nuffield Department of Medicine, University of Oxford, Oxford OX3 7BN, UK; 15 Mahidol–Oxford Tropical Medicine Research Unit (MORU), Mahidol University, Bangkok 10400, Thailand; 16 Department of Emergency Medicine, School of Medicine, Johns Hopkins University, Baltimore, MD 21205, USA; rrothma1@jhmi.edu; 17 Infectious Diseases, Intermountain Medical Center, Murray, UT 84107, USA; john.burke@imail.org; 18 School of Medicine, University of Utah, Salt Lake City, UT 84132, USA; 19 Department of Pharmacy Services, University of Chicago Medicine, Chicago, IL 60637, USA; gourang.patel@uchicagomedicine.org; 20 Infectious Disease, Loyola University Medical Center, Maywood, IL 60153, USA; jparada@lumc.edu

**Keywords:** sepsis, SeptiCyte RAPID, likelihood ratio, host-response biomarker, systemic inflammatory response syndrome, SeptiScore, diagnostic accuracy, blood culture, intensive care unit, gene expression

## Abstract

**Overview:** SeptiCyte RAPID is an FDA-cleared gene expression test that quantifies host immune response to aid in the diagnosis of sepsis. The test yields a score (the SeptiScore) ranging from 0–15, distributed across four bands (1–4) based on increased likelihood of sepsis. Each band can be characterized by average positive and negative likelihood ratios (LR+ and LR−, respectively) for the discrimination of sepsis versus the non-infectious systemic inflammatory response syndrome (SIRS). **Methods:** A retrospective analysis of prospectively collected data from a combined cohort of critically ill patients suspected of sepsis (*n* = 889), recruited across 19 hospitals in the USA and Europe. The analysis quantified the LR+ and LR− parameters as a function of SeptiScore, for discrimination of sepsis vs. SIRS in patients admitted to ICU. **Hypotheses:** (1) The likelihood ratio (LR) framework provides a clinically useful interpretive approach that complements the previously used SeptiScore banding scheme; (2) Low Band 1 SeptiScores are associated with sufficiently small LR− to support the use of SeptiCyte RAPID as a rule-out test for sepsis; (3) High Band 4 SeptiScores are associated with sufficiently large LR+ to support the use of SeptiCyte RAPID as a rule-in test for sepsis; and (4) SeptiScore-derived LR+ and LR− values can be combined with estimates of pre-test probability (derived from patient characteristics and/or other diagnostic tests) to generate individualized, patient-specific post-test probabilities of sepsis. **Results:** The SeptiCyte RAPID test demonstrates strong diagnostic performance in distinguishing sepsis from SIRS. The likelihood ratios across different score bands provide clear clinical utility: the median LR+ was 3.26 (range 2.57–4.24) for Band 3, and 6.97 (range 4.35–15.57) for Band 4, providing evidence toward ruling in sepsis at high SeptiScores. Conversely, the median LR− was 0.16 (range 0.14–0.20) for Band 2 and 0.085 (range 0.014–0.16) for Band 1, providing evidence toward ruling out sepsis at low SeptiScores. A higher-resolution analysis of SeptiCyte RAPID performance confirmed these trends by evaluating LR+ and LR− at specific values within each band. The sepsis group was further stratified according to whether patients were classified as blood culture positive (BC+) or blood culture negative (BC−), and the detailed LR+ and LR− analyses were repeated. A monotonic increase in likelihood ratio with increasing SeptiScore was consistently observed, independent of whether sepsis patients were culture-positive, culture-negative, or unstratified with respect to blood culture status. **Conclusions:** High SeptiScores have correspondingly high LR+ values, and low SeptiScores have correspondingly low LR− values, both of which may have clinical utility. High likelihood ratios for Band 4 SeptiScores, which precede traditional microbiology results, may provide clinicians with early confidence of a sepsis diagnosis and microbiology diagnostic stewardship. Low likelihood ratios for Band 1 SeptiScores may prompt clinicians to consider an alternate diagnosis to sepsis. These are diagnostic-performance-based observations; whether they translate into fewer missed diagnoses or more efficient use of hospital resources has not been directly assessed in this study and will require dedicated clinical outcome studies.

## 1. Introduction

SeptiCyte RAPID is an FDA-cleared real-time quantitative RT-PCR test that measures the relative expression levels of two host immune response genes, PLA2G7 and PLAC8, which are relevant to the diagnosis of sepsis [1]. The test yields a SeptiScore, equal to the difference in threshold cycle (Cq) values between the PLA2G7 and PLAC8 amplicons. The SeptiScore ranges from 0–15, with higher SeptiScores indicating an increased likelihood of sepsis. The SeptiScore range has been divided into four Bands (B1 SeptiScores 0–4.9; B2 SeptiScores 5.0–6.1; B3 SeptiScores 6.2–7.3; B4 SeptiScores 7.4–15, Supplementary Section S1) [1]. Each SeptiScore Band can be characterized by performance measures that are significantly affected by sepsis prevalence, such as negative and positive predictive values. SeptiScore Bands can also be characterized by prevalence-independent measures such as sensitivity, specificity, and band-specific likelihood ratios (LR+ and LR−).

The likelihood ratio (LR) concept provides a context-dependent framework for evaluating SeptiCyte performance by combining both sensitivity and specificity into a single, stable measure that quantifies how much a test result changes the likelihood of disease [2]. Unlike other performance measures, which describe test accuracy in isolation, LRs usefully integrate pre-test and post-test probabilities through Bayes’ theorem, thereby offering a direct measure of how test results can modify a clinician’s a priori estimate of disease probability. The use of LRs thus may enhance clinical decision-making by translating statistical accuracy into meaningful disease probability estimates that are relevant to patient care [3]. A range of other blood-based immune response biomarkers, including C-reactive protein (CRP), procalcitonin (PCT), interleukin-6 (IL-6), neutrophil CD64 expression, pancreatic stone protein (PSP), and, more recently, a microfluidic biophysical assay (Cytovale Intellisep), have also been proposed as aids to the diagnosis of sepsis. The reported likelihood ratios of these biomarkers are referenced below to provide context for SeptiCyte RAPID results.

This study describes a detailed LR analysis for SeptiCyte RAPID in a combined cohort of critically ill (ICU) patients suspected of sepsis (*n* = 889) recruited in 19 hospitals across the USA and Europe. Besides estimating LR+ and LR− for each SeptiScore Band, a higher-resolution analysis of SeptiCyte RAPID performance was also considered, in which the LR+ and LR− were evaluated across a continuous scale of SeptiScore values, rather than being restricted to the four pre-defined interpretation bands. A further stratification was also conducted, in which the sepsis patient group was stratified with respect to being blood culture positive (BC+) or blood culture negative (BC−), and the LR analysis repeated.

Four hypotheses are addressed in this study: (1) the likelihood ratio (LR) framework provides a clinically useful interpretive approach that complements the previously used SeptiScore banding scheme; (2) Low Band 1 SeptiScores are associated with sufficiently small negative likelihood ratios (LR−) to support the use of SeptiCyte RAPID as a rule-out test for sepsis; (3) High Band 4 SeptiScores are associated with sufficiently large positive likelihood ratios (LR+) to support the use of SeptiCyte RAPID as a rule-in test for sepsis; and (4) SeptiScore-derived LR+ and LR− values can be combined with estimates of pre-test probability (derived from patient characteristics and/or other diagnostic tests) to generate individualized, patient-specific post-test probabilities of sepsis.

## 2. Materials and Methods

### 2.1. Study Cohorts

**Figure 1:**
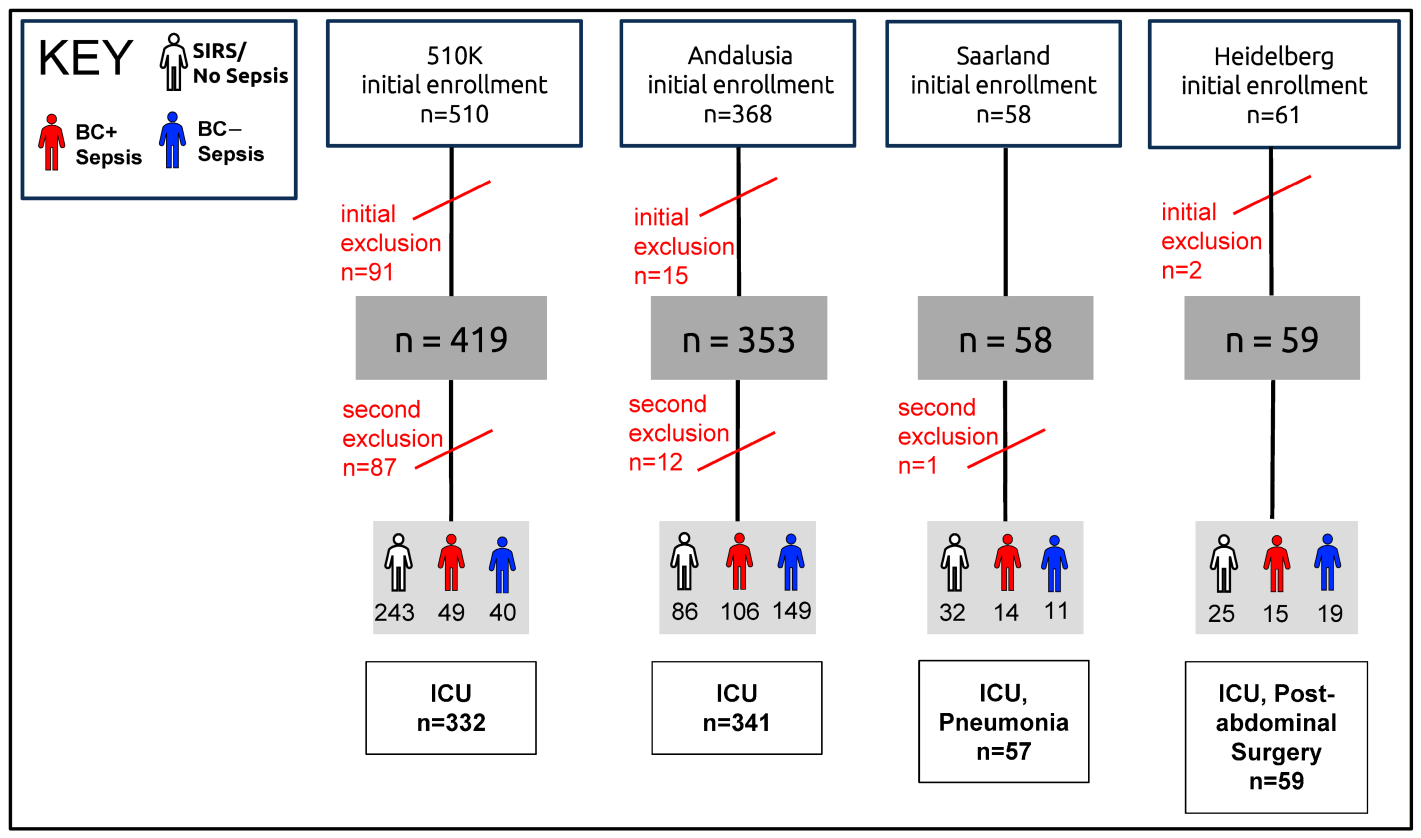
Consort diagram for sepsis vs. SIRS discrimination. Four cohorts were recruited across 19 sites. Total all sepsis = 503. Total BC (+) sepsis = 184. Total BC (−) sepsis = 219. Total SIRS = 386. Total for sepsis/SIRS analysis without BC stratification = 889. Total for sepsis/SIRS analysis with BC (+/−) stratification = 789.

Figure 1 presents a consort diagram describing the origins of the patients in this study. The patients were recruited from ICUs across 19 sites in Europe and the United States. Details of the individual patient cohorts in this study, including descriptions of all patient exclusions, are presented below. For all patients, blood was collected in PAXgene blood RNA tubes (FDA K042613; PreAnalytiX GmbH, Hombrechtikon, Switzerland).

**Immunexpress 510k cohort (final *n* = 419):** This cohort consisted of retrospective (*n* = 356) and prospective (*n* = 63) components. A full description of the study cohort and a flow diagram for the origin of all samples used in the study have previously been published [1]. The retrospective component of critically ill (ICU) patients was drawn from the observational MARS, VENUS, and VENUS Supplement trials (NCT01905033 and NCT02127502 on clinicaltrials.gov). The recruitment dates were January 2011–December 2013 (MARS), May 2014–April 2015 (VENUS), and March–August 2016 (VENUS Supplement). The prospective component consisted of 63 critically ill (ICU) adult subjects enrolled in an observational trial (NEPTUNE, NCT05469048; clinicaltrials.gov (accessed on 17 February 2024)) between the dates 26 May 2020 and 25 April 2021 at Emory University/Grady Memorial Hospital (Atlanta, GA, USA), Rush University Medical Center (Chicago, IL, USA) and University of Southern California (USC) Medical Center (with two separate sites, Keck Hospital of USC, and Los Angeles General Medical Center, in Los Angeles, CA, USA). The NEPTUNE inclusion/exclusion criteria matched the criteria for the earlier studies.

*initial enrolment:* 510 ICU patients
*first-stage exclusion:* 91 excluded because banked PAXgene blood sample no longer available
*second-stage exclusion:* 87 sepsis patients excluded because no BC (+/−) results documented
*n for unstratified analysis:* 419
*n for BC+/− analysis:* 332
  *243 SIRS*
  *49 BC* (+) *sepsis*
  *40 BC* (−) *sepsis*

*Comparator:* Classification as sepsis or SIRS was achieved through a process of retrospective physician diagnosis (RPD) by a panel of three expert clinicians not involved with the care of the patients. The method has been thoroughly described in Supplement 3 of Miller et al. (2018) [4]. If at least two out of the three panelists agreed that the patient had sepsis or SIRS, then that adjudication was accepted as Consensus RPD, leaving 41 (9.8%) indeterminate cases. Patients with an “indeterminate” call underwent an additional independent blinded case review and were forced into a binary classification of sepsis or SIRS. Under this secondary adjudication (“Forced RPD”), no indeterminate calls were allowed, lending access to the full cohort.

**Heidelberg cohort (*n* = 59):** The “SeptAsTERS” study was conducted at a single hospital in Germany. The final enrollment was of 59 post-surgical ICU patients with SIRS and showing signs of clinical deterioration. A total of 34 (57.6%) were retrospectively determined to have sepsis, and the remaining 25 (42.4%) were determined to have SIRS. Blood cultures were ordered on the basis of clinical judgement and accordingly were only taken if deemed necessary by the care team. Of the 34 septic patients, 15 (44.1%) were blood culture positive.

*Initial enrolment:* 61 ICU patients
*first-stage exclusion:* 2 patients excluded (1 readmission, and 1 palliative care)
*second-stage exclusion:* none
*n for unstratified analysis:* 59
*n for BC+/− analysis:* 59
  *25 SIRS*
  *15 BC (+) sepsis*
  *19 BC (−) sepsis*

*Comparator:* The comparator for classification as SIRS or sepsis in this study was a post hoc assessment of all patients by three independent intensive care professionals who were not involved in the study. The process followed a model similar to the one described in the online data supplement Part 3 by Miller et al. (2018) [4] based on FDA Guidance and on publications by Klein Klouwenberg et al. (2013) [5]. Details of the post hoc assessment process are provided in von der Forst et al. (2024) [6]. As organ dysfunction parameters were considered, the assessment can be viewed as having been performed under the Sepsis-3 conceptual framework [7].

**Andalusia cohort (*n* = 353)** (Cantón-Bulnes et al., 2026) [8]: An observational, prospective, and multicenter study was conducted between 3 March 2022 and 20 December 2022 in seven ICUs in Andalusia (Spain) and coordinated by the Virgen Macarena University Hospital in Seville. A total of 354 patients were enrolled, of whom 86 (24.3%) did not present sepsis at the researchers’ discretion. All patients aged 18 years or older, admitted to the ICU, with a diagnosis of sepsis, according to the Sepsis-3 definition, were included. Subjects were excluded if they were pregnant, or the clinical picture suggestive of sepsis had started more than 48 h previously.

*initial enrolment:* 368 ICU patients
*first-stage exclusion:* 15 patients excluded (8 consent not given; 2 satisfying exclusion criteria;

3 with analytical failures; 1 duplicate; 1 for non-consensual classification) (see Supplementary Figure S2 in Cantón-Bulnes et al., 2026 [8])

*second-stage exclusion:* 12 sepsis patients excluded because no BC (+/−) results documented
*n for unstratified analysis:* 353
*n for BC+/− analysis:* 341
  *86 SIRS*
  *106 BC (+) sepsis*
  *149 BC (−) sepsis*

*Comparator:* In a retrospective analysis, patients were categorized as “sepsis” or “sterile inflammation” (i.e., SIRS) by two investigators at each participating site who were aware of the clinical, analytical, and microbiological data but blinded to the SeptiCyte results. A steering committee reviewed all cases and contacted the site investigators in case of doubts.

**Saarland cohort (*n* = 58)** (Benthien et al. 2024) [9]: Patients aged ≥ 18 years at or admitted to the pneumological ICU with a suspicion of sepsis constituted the eligible study patient population. Patients aged < 18 years, pregnant patients, as well as the absence of written informed consent constituted cases that are not to be considered for study entry (exclusion criteria). In addition, patients who were on therapeutic antibiotic treatment for longer than 48 h prior to SeptiCyte RAPID sampling were also excluded.

Patients were recruited from the ICU at Pneumology and Intensive Care Medicine, Saarland University, Homburg, Germany. Between November 2022 and February 2024, a total of 39 symptomatic patients with a sepsis-like clinical pattern and 18 non-septic controls from the same ICU were recruited. Subjects were included if they had a change in the SOFA score within 24 h of ICU admission of ≥2 and antimicrobial therapy for ≤48 h. After having obtained informed consent, peripheral blood samples from the patients were collected using PAXgene RNA tubes (PreAnalytiX GmbH, Hombrechtikon, Switzerland) and subjected to the SeptiCyte RAPID assay. Clinicians were blinded to the SeptiCyte results.

*initial enrolment:* 58 ICU patients
*first-stage exclusion:* none
*second-stage exclusion:* 1 sepsis patient excluded because no BC (+/−) results documented
*n for unstratified analysis:* 58
*n for BC+/− analysis:* 57
  *32 “SIRS” (i.e., non-septic controls)*
  *14 BC (+) sepsis*
  *11 BC (−) sepsis*

*Comparator:* RPD was conducted with a panel of clinicians not involved in patient care. The RPD process led to a final clinical adjudication of 26 patients with infection-related sepsis and 32 patients with non-sepsis status, which are placed in the SIRS category.

### 2.2. Ethics Approval and Consent to Participate

All research reported in this study was conducted in accordance with the Declaration of Helsinki and in accordance with relevant guidelines and regulations. Informed consent was obtained from all subjects or their legal representatives.

*Heidelberg:* The SeptAsTERS component of this study was approved by the Ethics Committee of the Medical Faculty of Heidelberg University (S-118/2021) and was registered in the German Clinical Trials Register (DRKS00024891) prior to enrollment.

*MARS:* Ethics approval for the MARS trial was given by the Medical Ethics Committee of the Amsterdam Medical Canter (approval # 10-056C).

*VENUS:* Ethics approvals for the VENUS trial were given by the relevant Institutional Review Boards as follows: Intermountain Medical Center/Latter Day Saints Hospital (approval # 1024931); Johns Hopkins Hospital (approval # IRB00087839); Rush University Medical Center (approval # 15111104-IRB01); Loyola University Medical Center (approval # 208291); Northwell Healthcare (approval #16-02-42- 03).

*NEPTUNE:* Ethics approvals for the NEPTUNE trial were given by the relevant Institutional Review Boards as follows: Emory University (approval # IRB00115400); Grady Memorial Hospital (approval # 00-115400); Rush University Medical Center (approval # 19101603-IRB01); University of Southern California Medical Center (approval # HS-19-0884-CR001).

*Andalusia:* The study was approved by the Research Ethics Committees of the Virgen Macarena-Virgen Rocio University Hospitals on 20 December 2021 (Internal Code, 2662-N-21). Since sepsis is a time-dependent process and the clinical outcome depends on how quickly it is recognized, this assay should be performed as soon as possible after clinical suspicion of sepsis. Based on this argument, the research ethics committees allowed blood sample extractions prior to obtaining written permission. Written consent from the patient or next of kin was obtained within 48 h of ICU admission, and the blood sample was discarded if written consent was not obtained. The study was approved by the Research Ethics Committees of the Virgen Macarena-Virgen Rocio University Hospitals (Certificate of Ethics Approval dated 23 December 2021. Trial name SEPT-ANC. Codigo Interno: 2662-N-21.)

*Saarland:* The study was approved by the Ethics Commission of the Saarland Medical Association for Saarland University (approval # 68/22, date 23 May 2022).

### 2.3. Statistical Methods

Calculations of the area under the receiver operating characteristic curve (AUC), sensitivity, and specificity were performed in R (version 4.3.1) using the *pROC* package (version 1.19.0.1), specifically the roc function, as described by Robin et al. (2011) [10]. To estimate likelihood ratios (LRs) across the full range of SeptiScore values, two complementary approaches were used to account for differences in data density.

(1) In the central region of the SeptiScore distribution, where observations were relatively abundant, LRs were derived from receiver operating characteristic (ROC) analysis using corresponding estimates of sensitivity and specificity at the relevant operating points:

LR^+^ = sensitivity/(1 − specificity) LR^−^ = (1 − sensitivity)/specificity (1)

(2) In the tails of the SeptiScore distribution where data were sparse, ROC-based estimates become unstable due to limited sample density. To improve statistical robustness in these tail regions, we binned SeptiScore values into intervals containing at least five patient observations per bin (≥5 samples per interval). Likelihood ratios (LR^+^ and LR^−^) were calculated empirically from the resulting 2 × 2 contingency tables constructed within each interval. The relationship between LR+, LR− and the contingency table values of true positives (TP), false positives (FP), true negatives (TN), and false negatives (FN) is given by the following derivation:

sensitivity = TP/(TP + FN) specificity = TN/(TN + FP)(2)

LR^+^ = (TP/(TP + FN))/(FP/(FP + TN)) LR^−^ = (FN/(TP + FN))/(TN/(FP + TN)) (3)

LR+ = (TP/FP) * (TN+FP)/(TP+FN) LR− = (FN/TN) * (TN+FP)/(TP+FN)(4)

For Tables 2–5 below, each SeptiScore Band’s overall LR+ or LR− (minimum, median, mean, maximum) was computed from the set of pointwise LR+/LR− estimates falling within that Band’s SeptiScore range (Equations (1)–(4)). The 95% CI reported alongside each Band’s mean was estimated as mean ± t (0.975, *n* − 1) × SE, where n is the number of pointwise estimates contributing to that Band’s average and SE is their standard error, i.e., a one-sample t-distribution-based confidence interval for the mean [11]. At the upper bound of Band 4, pointwise LR+ estimates where specificity = 1 (making LR+ mathematically undefined) were excluded from the mean, 95% CI, and maximum.

**Figure 2:**
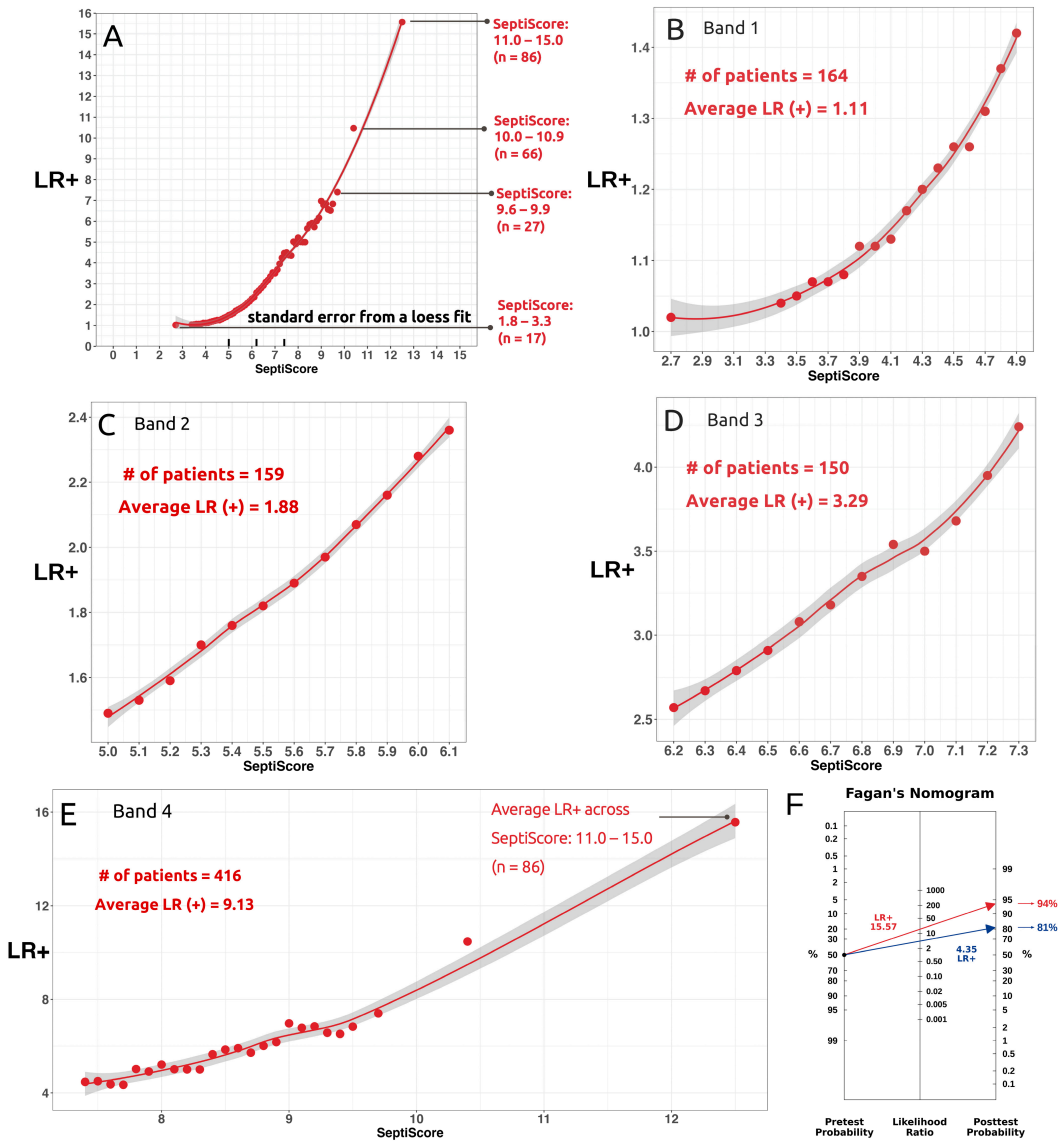
Positive likelihood ratio (LR+) analysis of complete dataset (*n* = 889). No stratification by BC (+/−) was performed. (**A**) LR+ versus SeptiScore, over entire SeptiScore region. (**B**) Expanded view of Band 1 region. (**C**) Expanded view of Band 2 region. (**D**) Expanded view of Band 3 region. (**E**) Expanded view of Band 4 region. (**F**) Fagan nomogram for SeptiScore Band 4, assuming a pre-test sepsis prevalence of 50% and LR+ values of 4.35 and 15.57 (These LR+ values correspond to the Band 4 lower and upper bounds, respectively).

**Figure 3:**
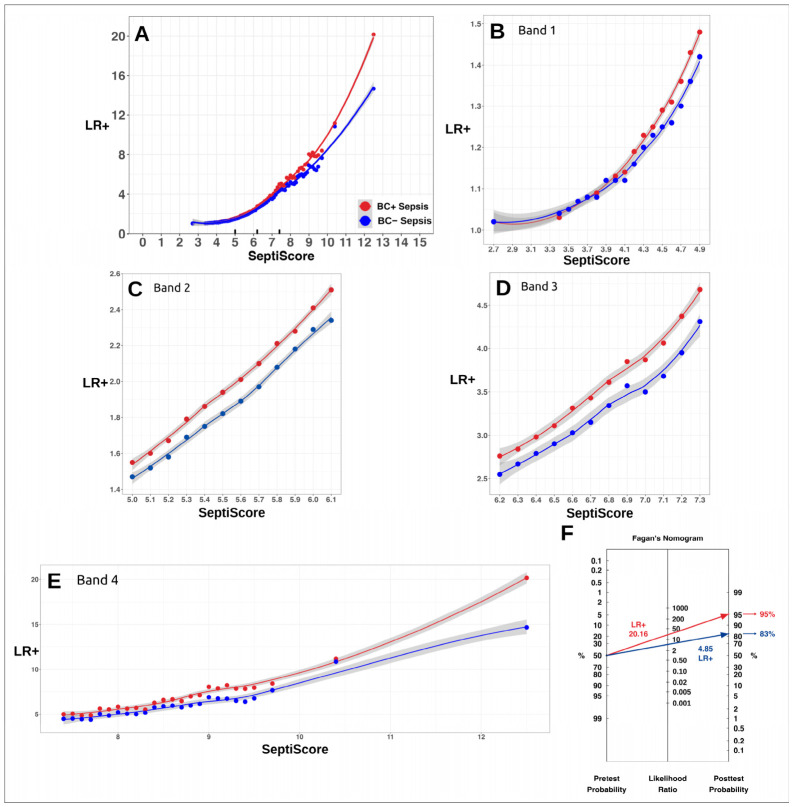
LR+ analysis for BC+ sepsis vs. SIRS, and for BC− sepsis vs. SIRS. (**A**) LR+ for 184 BC (+) sepsis vs. 386 SIRS (red line), and LR+ for 219 BC (−) sepsis vs. 386 SIRS (blue line). The X-axis shows shorter bars at 5.0, 6.2 and 7.4 which are the SeptiCyte RAPID Interpretation Band thresholds. (**B**) Expanded view of Band 1. (**C**) Expanded view of Band 2. (**D**) Expanded view of Band 3. (**E**) Expanded view of Band 4. For panels (**A**–**E**), the LOESS best fit lines and 95% error envelopes are shown. (**F**) Fagan nomogram for SeptiScore Band 4, assuming a pre-test sepsis prevalence of 50% and LR+ values of 4.85 (blue line) and 20.16 (red line) (these LR+ values correspond to the Band 4 lower and upper bounds, respectively).

**Figure 4:**
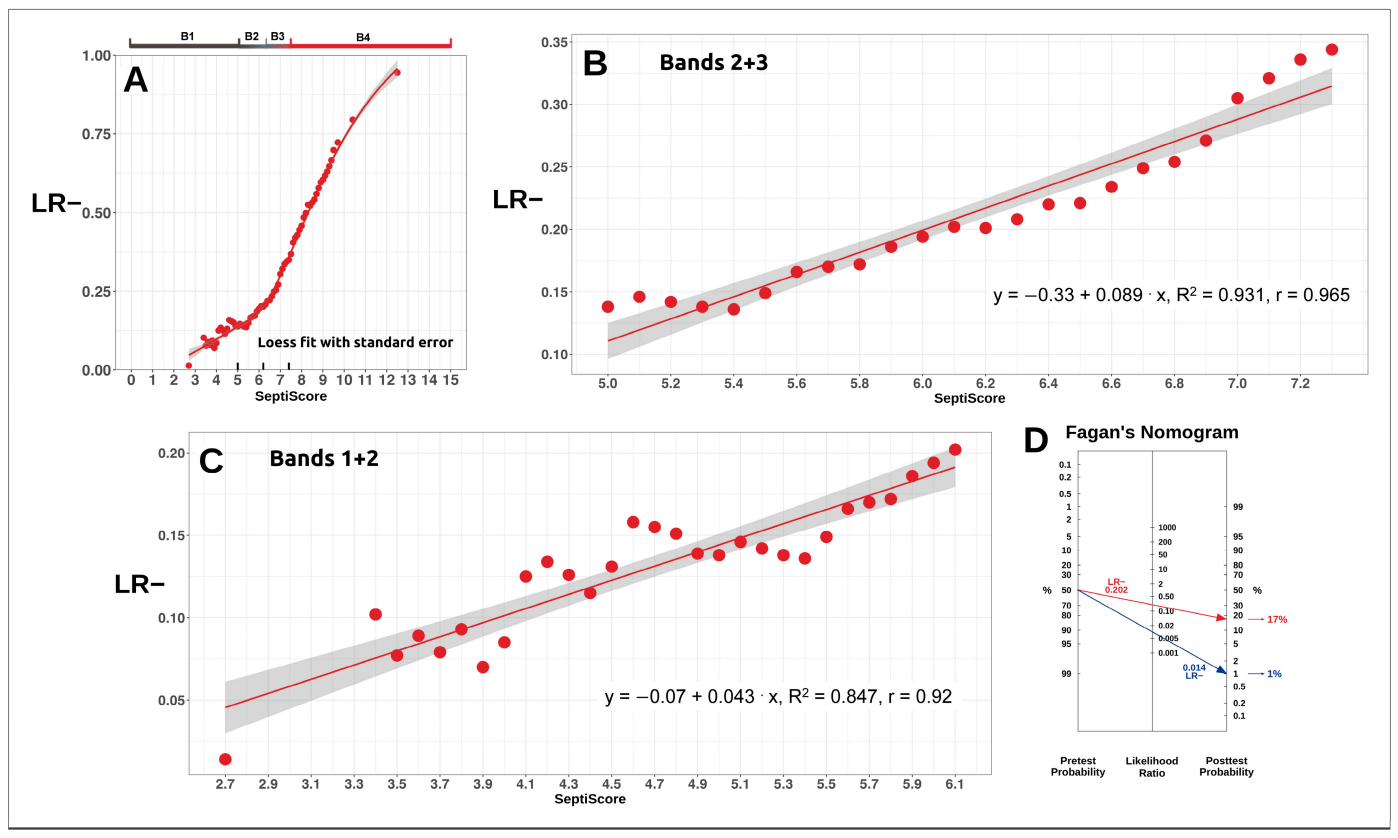
Negative likelihood ratio analysis. (**A**) LR− for all sepsis vs. SIRS (4 cohorts, *n* = 889) The X-axis shows shorter bars at 5.0, 6.2 and 7.4 which are the SeptiCyte RAPID Interpretation Band thresholds. (**B**) Expanded region of LR− plot for SeptiScore Bands 2 + 3. (**C**) Expanded region of LR− plot for SeptiScore Bands 1 + 2. (**D**) Fagan’s nomogram for LR− SeptiScore Bands 1 + 2, showing the shift in sepsis probability from pre-test (*p* 0.50) to post-test (*p* 0.17 or 0.01) for LR− 0.202 (red line) and 0.014 (blue line), respectively.

**Figure 5:**
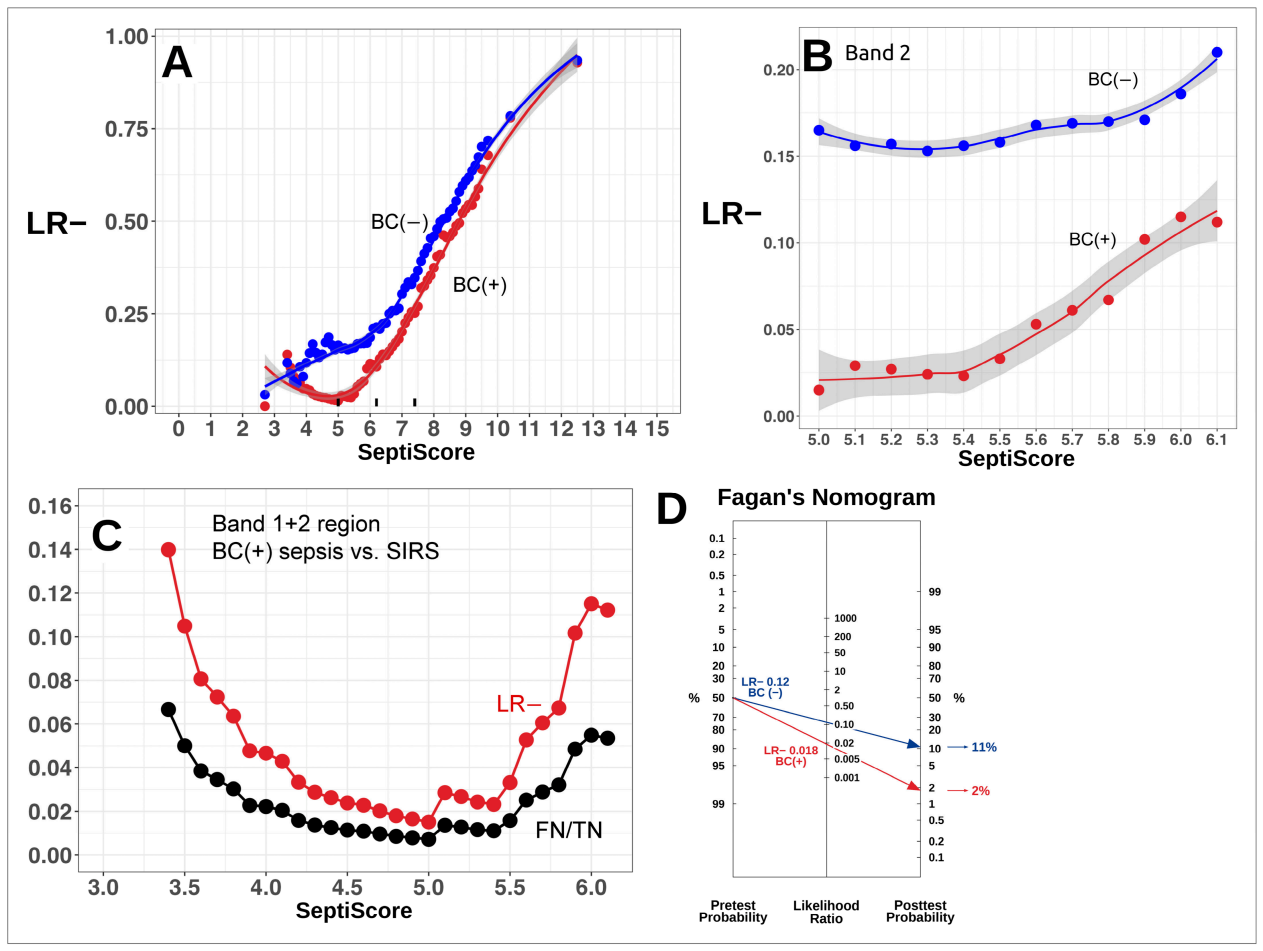
Negative likelihood ratio (LR−) stratified according to BC+ or BC− status. (**A**) Red: LR− for BC (+) sepsis (*n* = 184) vs. SIRS (*n* = 386). Blue: LR− for BC (−) sepsis (*n* = 219) vs. SIRS (*n* = 386). The X-axis shows shorter bars at 5.0, 6.2 and 7.4 which are the SeptiCyte RAPID Interpretation Band thresholds. (**B**) Expanded Band 2 region of LR− plot. (**C**) BC (+) sepsis vs. SIRS comparison, expanded Band 1–2 region, showing the “J-shape” behavior (see Supplementary Section S5). Note that this apparent instability reflects extremely small sample sizes in this region: only two BC (+) sepsis patients have SeptiScores below 5.0 (at 3.3 and 5.0), so that a single patient dominates the FN/TN ratio underlying the LR− calculation once the cutoff falls below 5.0 (Supplementary Section S5). (**D**) Fagan’s nomogram at LR− minima, assuming pre-test sepsis probability 50%. For BC (−), the minimum value LR− = 0.12 occurs at SeptiScore 3.4 (blue line in Panel (**A**)). For BC (+), the minimum value LR− = 0.018 occurs at SeptiScore 5.0 (red line in Panel (**A**)).

The positive and negative likelihood ratios (LR+ and LR−) were plotted as functions of SeptiScore. A smoothed best-fit curve was generated using local polynomial regression (LOESS) as implemented in the geom_smooth function of the R ggplot2 package (version 3.5.2). In the figures below, the gray shaded band surrounding the LOESS curve represents the 95% confidence interval for the fitted relationship. This confidence interval is derived from the standard errors of the LOESS fit, assuming these errors follow an approximate Student’s t distribution. The exact number of sepsis and SIRS patients contributing to each interval, including the extreme tail intervals shown in Figures 2–5 below, is provided in full in Supplementary Table S2 (unstratified analysis), Table S3 (BC (+) analysis), and Table S4 (BC (−) analysis).

The LOESS fits of LR+ and LR− as functions of SeptiScore used the default “geom_smooth” parameters (span = 0.75; local quadratic fitting, degree = 2; Gaussian weighting family), which were not modified from their software defaults. We recognize that the choice of smoothing window or span can influence the shape of the fitted curve, particularly in the tails of the SeptiScore distribution where data are sparse; a narrower span would track local fluctuations more closely at the cost of increased variance, whereas a wider span would yield a smoother but potentially more biased curve. The LOESS curves in Figures 2–5 should therefore be interpreted as descriptive smooths of the pointwise LR estimates rather than as validated parametric models. The underlying tabulated LR values (Supplementary Tables S2–S4) are provided so that readers may apply alternative smoothing approaches if desired.

To assess the consistency of LR estimates across the four component cohorts, a cohort-level heterogeneity analysis was performed at each of the three pre-specified SeptiScore Band-boundary cutoffs (5.0, 6.2, 7.4). At each cutoff, cohort-specific LR+ and LR− were computed from cohort-specific 2 × 2 contingency tables, with log-scale standard errors derived following Simel et al. (1991) [12] and Deeks and Altman (2004) [3]. Cohort-specific estimates were then pooled via a DerSimonian and Laird (1986) [13] random-effects meta-analysis, and heterogeneity was quantified using Cochran’s Q and the I^2^ statistic. I^2^ was calculated as I^2^ = 100 × (Q − df)/Q, where Q is Cochran’s Q statistic and df is its degrees of freedom (number of cohorts minus one) [14,15]. As a threshold-free complement to this analysis, a logistic regression model of sepsis status on SeptiScore with a SeptiScore-by-cohort interaction term was compared, via likelihood ratio test, to the corresponding main-effects-only model [16]. Full methodological detail, per-cohort results, and forest plots are provided in Supplementary Section S7.

As a visualization aid, we used a custom R program to construct Fagan nomograms in log-odds space to represent the relationship between pre-test probability, likelihood ratio (LR), and post-test probability. In this space, the post-test log-odds is the sum of the pre-test log-odds and the log of the LR. To ensure that lines connecting pre-test probabilities to post-test probabilities via the LR axis appear straight, the post-test axis was affine-transformed (both shifted and rescaled) relative to the pre-test axis. This adjustment preserves the additive property of log-odds and allows multiple LRs to be plotted simultaneously, providing an accurate visual representation of post-test probabilities [17].

## 3. Results

### 3.1. Patient Cohorts

We recruited a total of 889 intensive care patients comprising four cohorts from 19 hospital sites in Europe and the USA. Data from 789 of the patients were available for the BC +/− analysis. In total, 184 BC (+) sepsis patients, 219 BC (−) sepsis patients, 100 sepsis patients without BC data, and 386 SIRS patients were included in the analyses. Figure 1 in Materials and Methods presented a flow diagram showing patient numbers from each study that were included or excluded, and the number of patients retrospectively determined to be sepsis or SIRS, or BC+/BC− sepsis. Table 1 below summarizes some basic demographic data for these patient cohorts. Although the patients were all recruited from the ICU, considerable diversity is evident with respect to race/ethnicity, age, and mortality rate.

### 3.2. Positive Likelihood Ratios (LR+)

Table 2 and Figure 2 show, for the entire available dataset (4 cohorts, *n* = 889), the relationship between the SeptiScore and the positive likelihood ratio (LR+). There is a clear monotonic increase in LR+ as a function of increasing SeptiScore. In Table 2, the 95% CI reported for the mean LR+ in each Band was computed via the t-distribution across the set of pointwise LR+ estimates contributing to that Band’s average, as described in Section 2.3 of Materials and Methods. The 95% confidence envelope for the pointwise LR+ estimate at each SeptiScore value is shown graphically as the grey band surrounding the LOESS curve in Figure 2.

**Table 2:** Positive likelihood ratios for sepsis/SIRS discrimination in the complete dataset (*n* = 889). The minimum (min), maximum (max), median, and mean LR+ values for each SeptiScore Band were estimated from the distributions provided in Supplementary Table S2.

SeptiScore Band	Range	LR+ Min, or at Lower Boundary	Median LR+	Mean LR+	95% CI LR+	LR+ Max, or at Upper Boundary	*n* Sepsis	*n* SIRS	*n* Total
1	0.0–4.9	1.00	1.07	1.11	1.07–1.16	1.42	25	139	164
2	5.0–6.1	1.49	1.86	1.88	1.70–2.07	2.36	42	117	159
3	6.2–7.3	2.57	3.26	3.29	2.96–3.62	4.24	81	69	150
4	7.4–15.0	4.35	6.97	9.13	7.50–10.77	(average for SeptiScore 11–15: 15.57)	355	61	416
totals							503	386	889

In Figure 2, Panel A shows a graph of LR+ versus SeptiScore over the entire SeptiScore range, for the complete dataset of 503 sepsis vs. 386 SIRS patients. Panels B, C, D, and E show expanded views of the Band 1, 2, 3, and 4 regions, respectively. In these panels, the 95% uncertainty cloud around the LOESS best fit line is shown in grey. As an illustrative example, Panel F shows the Fagan nomogram corresponding to the low (4.35) and high (15.57) LR+ values for SeptiScore Band 4, assuming a pre-test sepsis probability of 50%. Note: in this and subsequent nomograms, the post-test probability axis has been affine transformed (shifted and stretched) relative to the pre-test probability and LR axes, so as to present the pre-test/post-test relationships as straight lines on this type of plot.

Table 3 and Figure 3 stratify the above analysis according to whether, in the comparison to SIRS, the septic patients could be classified as BC (+) or BC (−). A total of 100 sepsis patients without reported BC data were excluded in this secondary analysis. As in Table 2, the corresponding pointwise 95% confidence envelopes are displayed graphically around the LOESS curves in Figure 3. The 95% CI shown for each mean LR+ was computed via the t-distribution across the pointwise LR+ estimates contributing to that Band’s average, separately for the BC (+) and BC (−) comparisons.

**Table 3:** LR+ analysis per SeptiScore Band, stratified according to whether BC (+) sepsis or BC (−) sepsis is being compared to SIRS. For this secondary analysis, 789 patients were used. The minimum (min), maximum (max), median, and mean LR+ values for each SeptiScore Band were estimated from the distributions provided in Supplementary Tables S3 and S4.

SeptiScore Band	SeptiScore Range	Median LR+ for BC+ Sepsis vs. SIRS	Mean LR+ for BC+ Sepsis vs. SIRS	95% CI (BC+)	Median LR+ for BC− Sepsis vs. SIRS	Mean LR+ for BC− Sepsis vs. SIRS	95% CI (BC−)	No. of Patients
1	0.0–4.9	1.07	1.13	1.07–1.19	1.07	1.11	1.06–1.16	153
2	5.0–6.1	1.97	1.99	1.79–2.19	1.85	1.88	1.69–2.07	147
3	6.2–7.3	3.52	3.57	3.18–3.96	3.24	3.29	2.94–3.63	128
4	7.4–15.0	8.04	10.41	8.34– 12.48	6.90	9.02	7.38– 10.65	361

In Figure 3, Panel A shows the LR+ vs. SeptiScore relationship for 184 BC (+) sepsis vs. 386 SIRS (red line), and also the analogous relationship for 219 BC (−) sepsis vs. 386 SIRS (blue line). In both cases, the LR+ increases monotonically with increasing SeptiScore, indicating that elevated SeptiScores are associated with greater likelihoods of sepsis. Panels B, C, D, and E show expansions of the regions within Bands 1, 2, 3, and 4, respectively. It is evident from the close-ups shown in these panels that the BC (+) sepsis vs. SIRS curve falls above the BC (−) sepsis vs. SIRS curve for all SeptiScores ≥ 4.0. Panel F shows a Fagan nomogram for Band 4 BC (+) sepsis vs. SIRS, assuming a pre-test probability of 50% and using the minimum (4.85) and maximum (20.16) LR (+) values across this Band.

### 3.3. Negative Likelihood Ratios (LR−)

Table 4 and Figure 4 explore, for the entire available sample set of sepsis and SIRS/no sepsis patients (4 cohorts, *n* = 889), the relationship between the SeptiScore and the negative likelihood ratio (LR−). No stratification according to BC (+/−) was performed. Table 4 summarizes the LR− values at the lower and upper boundaries of each band, and also the mean and median LR− values for each band. The corresponding pointwise 95% confidence envelopes are displayed graphically around the LOESS curves in Figure 4. The 95% CI shown for each mean LR− was computed via the t-distribution across the pointwise LR− estimates contributing to that Band’s average.

**Table 4:** LR− analysis for sepsis/SIRS discrimination, per SeptiScore Band. The dataset consists of 889 patient samples, without stratification by BC (+/−) status. The minimum, maximum, mean, and median LR− values for each SeptiScore Band were estimated from the distributions provided in Supplementary Section S1.

Band	Range	LR− Min, or at Lower Boundary	Median LR−	Mean LR−	95% CI LR−	LR− Max, or at Upper Boundary	*n*
1	0.0–4.9	0.014	0.085	0.073	0.050–0.096	0.158	164
2	5.0–6.1	0.136	0.16	0.16	0.147–0.177	0.202	159
3	6.2–7.3	0.201	0.25	0.26	0.231–0.296	0.344	150
4	7.4–15.0	0.349	0.81	0.76	0.714–0.813	0.944	416

Figure 4 explores the relationship between SeptiScore and the negative likelihood ratio (LR−) across the SeptiScore range. All septic patients were included: there was no stratification according to whether the septic patients were BC (+), BC (−), or BC unknown. As shown in Panel A for a total of 503 sepsis vs. 386 SIRS patients, the LR− decreases monotonically with decreasing SeptiScore. Low SeptiScores are associated with markedly lower likelihoods of sepsis. Panel B shows an expanded region of the LR− versus SeptiScore plot for SeptiScore Bands 2 + 3. Panel C shows an expanded region of the LR− versus SeptiScore plot for SeptiScore Bands 1 + 2. Panel D shows Fagan’s LR− nomogram for SeptiScore Bands 1 + 2, assuming pre-test sepsis probability 50% and using the low (0.014, blue line) and high (0.202, red line) LR− values across these bands.

Table 5 and Figure 5 stratify the above analysis according to whether the septic patients can be classified as BC (+) or BC (−). A total of 100 sepsis patients without reported BC data have been excluded from this analysis. The corresponding pointwise 95% confidence envelopes are displayed graphically around the LOESS curves in Figure 5. The 95% CI shown for each mean LR− was computed via the t-distribution across the pointwise LR− estimates contributing to that Band’s average, separately for the BC (+) and BC (−) comparisons.

**Table 5:** LR− analysis per SeptiScore Band, stratified according to whether BC (+) sepsis orBC (−) sepsis is being compared to SIRS. For this analysis, 789 patient samples were used, consisting of 184 Sepsis BC (+), 219 Sepsis BC (−), and 386 SIRS.

SeptiScore Band	SeptiScore Range	LR− for BC+ Sepsis vs. SIRS				LR− for BC− Sepsis vs. SIRS			
		Median	Mean	95% CI	*n*	Median	Mean	95% CI	*n*
1	0.0–4.9	0.022	0.030	0.016–0.045	140	0.107	0.09	0.060–0.117	152
2	5.0–6.1	0.043	0.055	0.032–0.078	129	0.167	0.168	0.158–0.179	135
3	6.2–7.3	0.166	0.175	0.145–0.205	95	0.258	0.266	0.237–0.295	102
4	7.4–15.0	0.745	0.695	0.634–0.755	206	0.755	0.730	0.677–0.784	216
		total			570				605

Figure 5 explores further the relationship between SeptiScore and the negative likelihood ratio (LR−), after stratifying by whether BC (+) sepsis or BC (−) sepsis is being compared to SIRS. Note that in the BC (+) case, we observe a distinctive *J-shaped* curve with a minimum near 5.0 and an increasing trend as the SeptiScore decreases from 5.0 to 3.4 (red points in Figure 5A,C). This trend is mirrored by the FN/TN ratio (black points in Figure 5C) and is readily explained as a consequence of small sample size in the Band 1 SeptiScore region (see Supplementary Section S5). Consequently, due to the paucity of sepsis BC (+) cases in Band 1, LR− estimates in Band 1 are not very precise.

## 4. Discussion

This study has addressed the previously stated four hypotheses. Our analyses show that the likelihood ratio (LR) framework can provide a clinically useful interpretive approach that complements the previously used SeptiScore banding scheme. Low Band 1 SeptiScores are associated with sufficiently small LR− to support the use of SeptiCyte RAPID as a rule-out test for sepsis. Band 2 SeptiScores have a median LR− of 0.16 (range 0.14–0.20), indicating a moderate decrease in the likelihood of sepsis. High Band 4 SeptiScores are associated with sufficiently large LR+ to support the use of SeptiCyte RAPID as a rule-in test for sepsis. Band 3 SeptiScores have a median LR+ of 3.26 (range 2.57–4.24), indicating an increase in the likelihood of sepsis per the interpretation of LR+ in Elkahwagy et al. (2024) [18]. SeptiScore-derived LR+ and LR− values can be combined with estimates of pre-test probability (derived from patient characteristics and/or other diagnostic tests) to generate individualized, patient-specific post-test probabilities of sepsis.

From a clinical standpoint, distinguishing true infection from colonization or sterile systemic inflammation remains a central diagnostic challenge: neither bacterial isolation nor molecular/genetic detection (e.g., pathogen-directed PCR or sequencing) by itself confirms that an organism is provoking a clinically significant host response, as opposed to merely being present. Host-response-based tests such as SeptiCyte RAPID instead characterize the patient’s immune response, aiming to capture the extent to which a clinical presentation reflects a genuine infection-driven process.

Applying likelihood ratio (LR) analysis to SeptiCyte RAPID enhances the clinical interpretation of the SeptiScore for discriminating sepsis from SIRS. Unlike simple sensitivity and specificity metrics, LRs provide a direct measure of how a specific test result modifies the clinician’s a priori estimated probability of disease in an individual patient. Positive likelihood ratios (LR+) quantify how much a high SeptiScore (in Band 3 or 4) increases the likelihood of sepsis, while negative likelihood ratios (LR−) indicate how much a low SeptiScore (in Band 1 or 2) decreases it. Of course, SeptiCyte RAPID should be considered an *aid* to diagnosing sepsis, and therefore SeptiScores must be taken into consideration alongside other laboratory test results and vital signs.

### 4.1. Clinical Implications of High LR+ Values

High positive likelihood ratios (LR+) for Band 4 SeptiScores, which precede traditional microbiology results, may provide clinicians with early confidence of a sepsis diagnosis, and may support microbiology diagnostic stewardship.

Band 3 SeptiScores had a median LR+ of 3.26, while Band 4 SeptiScores had a median LR+ of 6.97. At the upper end of Band 4, the LR+ had a value of 15.57 (Figure 2E). At SeptiScores ≥ 11.4, the LR+ was ≥10 for the sepsis/SIRS discrimination. These results provide strong evidence to rule in sepsis at high SeptiScores.

When stratified on blood culture results, some differences in LR+ were evident between the BC+ sepsis/SIRS and BC− sepsis/SIRS comparisons. For the BC+ case, Band 3 and 4 SeptiScores had median LR+ of 3.52 and 8.04, respectively, while for the BC− case, the corresponding values were 3.24 and 6.90. For SeptiScores > 11.4, the LR+ was > 10 for BC+ sepsis. High SeptiScore values therefore may suggest that a blood culture be drawn prior to antibiotics being started. These BC− stratified comparisons should nonetheless be interpreted with caution. Blood culture positivity is influenced by multiple factors besides the severity of the host response, including the causative pathogen, prior antibiotic exposure, blood culture sampling technique and volume, and circulating bacterial load. These confounding factors may well contribute to the observed LR differences between BC (+) and BC (−) sepsis patients. However, the consistent direction of the effect across Bands 3 and 4 (higher LR+ in BC (+) sepsis) is also consistent with a genuine difference in host-response severity between microbiologically confirmed and culture-negative sepsis.

### 4.2. Clinical Implications of Low LR− Values

For sepsis/SIRS discrimination in Band 1, the median LR− was 0.014 irrespective of BC status, 0.022 for BC+ sepsis and 0.107 for BC− sepsis, respectively. These results indicate, in general, a very low probability of sepsis associated with Band 1 SeptiScores. Low LR− values for Band 1 SeptiScores may prompt clinicians to consider alternate diagnoses to sepsis and might also lead to better antibiotic stewardship. These potential downstream benefits are plausible extrapolations from the observed diagnostic likelihood ratios but were not directly measured in this study; prospective evaluation of clinical management and resource-utilization outcomes following adoption of LR−based interpretation is needed before such claims can be considered established.

A related consideration is the potential influence of prior antibiotic exposure on SeptiCyte RAPID performance, particularly among sepsis patients with negative blood cultures. Antibiotic pre-treatment can reduce or eliminate culture positivity even in genuinely septic patients, meaning that a proportion of BC (−) sepsis cases in this cohort may represent partially treated bloodstream infections rather than a distinct biological phenotype. We examined antibiotic-timing data, where available, in three of the four cohorts and found no evidence that antibiotic-exposure timing measurably shifted SeptiScore within BC (+) or BC (−) sepsis groups. This appears most consistent with SeptiCyte RAPID measuring host transcriptional response rather than pathogen viability. Full per-cohort results are provided in Supplementary Section S8.

### 4.3. Comparing SeptiCyte RAPID to Other Blood-Based Immune Response Sepsis Biomarkers

To provide further context, Table 6 and Table 7 summarize LR+ and LR− data from the literature for other blood-based immune response biomarkers that have been used as aids in sepsis diagnosis. These comparative tables demonstrate that the performance of SeptiCyte RAPID is relatively strong at both the high end (LR+) and the low end (LR−) of the 0–15 SeptiScore range. The comparative LR values in these tables are drawn from independent studies having different patient populations, clinical settings, disease prevalences, and reference standards for sepsis diagnosis. While this analysis helps to place SeptiCyte RAPID within a larger context, we recognize that it cannot substitute for a well-controlled prospective head-to-head comparison between SeptiCyte RAPID and alternative biomarkers. This is particularly relevant for widely used conventional biomarkers such as CRP and procalcitonin, whose levels rise only after a delay of several hours following infection onset [19,20] and can remain elevated in a variety of non-infectious conditions [21,22], limiting their usefulness for very early diagnosis or rapid rule-out decisions.

### 4.4. Applying Likelihood Ratio Analysis to Individual Patients

In applying likelihood-ratio-based analysis of sepsis versus SIRS to individual patients, a key parameter is the pre-test probability, which must be specified *a priori* and directly influences the resulting post-test probability estimates. In diagnostic terms, this represents the estimated likelihood that a patient has sepsis at the time of initial clinical assessment, prior to incorporating additional test results.

Our analysis estimates sepsis likelihood at the cohort level, using aggregated data to derive both pre-test probabilities and likelihood ratios (LRs). In clinical practice, however, clinicians typically assess sepsis risk on an individual basis, incorporating patient-specific factors such as age, comorbidities, vital signs, laboratory findings, and other contextual information. For likelihood-ratio-based (Bayesian) approaches to be clinically informative, the pre-test probability should therefore be individualized rather than population-based.

In the absence of detailed patient-level data, the pre-test probability may be approximated by the point prevalence of sepsis in the source population. Accordingly, we assumed an average prevalence of 50% ± 2.5% across all clinical sites in our study (Supplementary Section S6). This estimate exceeds the range reported in the ICON audit [33], suggesting the possibility of selection bias in our cohort; this limitation is discussed further below.

Recent studies have demonstrated approaches to estimating patient-specific sepsis risk using multivariable prediction models. For example, nomogram-based models integrating clinical features and comorbidities have been developed for general ICU populations [34], for patients using routinely collected vital signs and laboratory data [35], for urinary tract infection-associated sepsis [36], and for community-acquired pneumonia [37].

These tools illustrate how individualized risk estimation can provide a practical foundation for integrating likelihood-ratio-based reasoning into clinical decision-making. Such models could be extended to incorporate SeptiCyte RAPID scores, enabling a combined approach to estimating sepsis probability. Development of this integrated framework is the subject of ongoing work.

### 4.5. Limitations

We have identified some limitations to this study. First, the analysis is restricted to ICU patients. Although the cohort includes patients from 19 hospitals, the generalizability of these findings to patients presenting in the emergency department or hospital wards has not been established. In addition, the racial and ethnic composition of the cohort is uneven, with overrepresentation of White patients (67%) and relatively low representation of Hispanic and Asian patients (3.5–3.6% each). The available sample size does not support reliable, stratified analyses by race or ethnicity. A second limitation concerns study design. Although the four sub-studies collected data prospectively, none was originally designed with prespecified acceptance criteria for the likelihood ratios (LR+ and LR−) as functions of the SeptiScore. Consequently, the present analysis is best regarded as a retrospective (post hoc) analysis of prospectively collected data. Confirmation in a larger, purpose-designed prospective cohort with prespecified acceptance criteria would be an important next step.

Third, the adjudication process varied across cohorts (Section 2.1). The 510k and Saarland cohorts used retrospective physician diagnosis by an independent expert panel, the Heidelberg cohort used post hoc assessment under a Sepsis-3 framework, and the Andalusia cohort used site-investigator classification with steering committee review of uncertain cases. These methodological differences may influence the calculated likelihood ratios and cannot be fully corrected for retrospectively. On the other hand, this diversity mirrors real-world variation in how sepsis is diagnosed across institutions, so our pooled LR estimates should be interpreted as pragmatic, real-world estimates rather than idealized values obtained under a single uniform reference standard. Our cohort-level heterogeneity analysis (Section 4.2, Supplementary Section S7) determined that the LR estimates were highly consistent across cohorts at the primary rule-out threshold, and that the overall SeptiScore-sepsis relationship did not differ significantly by cohort. This strongly suggests our pooled estimates were not materially confounded by these differing adjudication approaches.

Fourth, the group of patients classified as SIRS is clinically heterogeneous. Although all met the inclusion criterion of at least two SIRS features, patients varied widely in age, comorbidities, and other clinical characteristics. This heterogeneity may introduce additional variability into the analysis; however, it also provides a degree of robustness, as likelihood-ratio-based performance appears to be maintained across a diverse patient population.

Fifth, estimates of the pre-test probability of sepsis (operationalized here as cohort-specific prevalence) vary substantially across our cohorts. Overall sepsis prevalence is approximately 42% in the 510k cohort, compared with approximately 75% in the Andalusian cohort. This heterogeneity likely reflects a combination of selection bias, differences in sepsis versus SIRS adjudication criteria, and variation in baseline illness severity across component sites within our cohorts.

Sixth, relatively few patients are represented at the extremes of the SeptiScore distribution. As a result, likelihood ratios (LRs) in these regions were estimated using interval-based approaches rather than point estimates, leading to reduced resolution and wider confidence intervals at the tails of the LR versus SeptiScore relationship. We note, however, that the Band-level patient distribution does not show a concentration in the middle of the range. Band 1 and Band 4 together account for 65.2% of the cohort (164/889 and 416/889 patients, respectively), consistent with the expected bimodal separation between SIRS and sepsis (Band 1: 36.0% of SIRS vs. 5.0% of sepsis; Band 4: 15.8% of SIRS vs. 70.6% of sepsis). The reduced resolution instead reflects sparse individual SeptiScore values near the numeric extremes of the 0–15 range, not a lack of patients in the outer Bands.

Finally, this analysis has pooled data from four cohorts that differ in geography, recruitment era, inclusion criteria, and sepsis/SIRS adjudication methodology (Section 2.1). Likelihood ratios are much less affected by disease prevalence than are predictive values, but they remain susceptible to spectrum effects, so we formally assessed cohort-level heterogeneity using the patient-level dataset. At each of the three pre-specified SeptiScore Band-boundary cutoffs (5.0, 6.2, 7.4), cohort-specific LR+ and LR− were computed and pooled via DerSimonian–Laird random-effects meta-analysis (Supplementary Section S7, Figure S7A–C). At the Band1/2 boundary (5.0, our rule-out threshold), cohort-specific LR− estimates were highly consistent (I^2^ = 5%, pooled LR− = 0.16, 95% CI 0.11–0.25). At the Band3/4 boundary (7.4, our rule-in threshold), cohort-specific LR+ estimates showed moderate heterogeneity (I^2^ = 53%, pooled LR+ = 3.57, 95% CI 2.49–5.14), but the *p*-value from Cochran’s Q test = 0.094 was not statistically significant. Additionally, a threshold-free logistic-regression interaction test was not statistically significant (*p* = 0.153). (The reader may refer to Supplementary Section S7 for complete per-boundary statistics and forest plots.) Taken together, these results support the consistency of the rule-out threshold across cohorts, while indicating that the rule-in LR+ magnitude should be generalized with some caution.

## 5. Conclusions

The foregoing analysis suggests that a likelihood-ratio-based re-interpretation of SeptiScore results may usefully complement the existing four-band classification scheme, by providing a transparent, quantitative link between a patient’s pre-test probability of sepsis and a personalized post-test probability. High Band 4 SeptiScores, with LR+ values that can exceed 10 above a SeptiScore of 11.4, may support earlier clinical confidence in a sepsis diagnosis and targeted microbiology stewardship. Low Band 1 SeptiScores, with correspondingly low LR− values, may support alternate, non-infectious diagnoses. For clinical practice, we suggest that SeptiScore-derived likelihood ratios be used as one consideration among several in the overall assessment of a critically ill patient, rather than as a stand-alone rule-in or rule-out criterion, and that any protocol incorporating SeptiScore-based likelihood ratios into antibiotic or diagnostic decision-making be developed and validated locally.

Important limitations of the present analysis (detailed above) imply that these conclusions should be regarded as hypothesis-generating with respect to clinical management, even though they are well supported with respect to confirming diagnostic performance. Larger, prospectively designed, multi-center studies that directly evaluate clinical outcomes (including antibiotic use, time to appropriate therapy, and missed or delayed sepsis diagnoses) associated with SeptiScore-derived likelihood ratio interpretation are needed before this framework can be recommended for routine clinical decision-making.

## Figures and Tables

**Table 1:** Demographics, including either all patients (*n* = 889), or after excluding * sepsis patients for whom no BC results were available (*n* = 789). Abbreviations: A (Asian), B (Black), H (Hispanic), W (White), O (other/unknown), *n*/A (data not available).

Cohort	*n*	*n* Male (%)	Age: Median (Range)	Race/Ethnicity	*n* Sepsis (%)	*n* (%) Died in Hospital
510 k	419	232 (55.4%)	59 (18–90)	W 254 (60.6%) B 115 (27.5%) H 22 (5.2%) A 21 (5.0%) O 7 (1.7%)	176 (42.0%)	44 (10.5%)
	or 332 *	187 (56.3%)	58 (18–90)	W 204 (61.5%) B 90 (27.1%) H 17 (5.1%) A 16 (4.8%) O 5 (1.5%)	89 (26.2%)	30 (9%)
Andalusia	353	219 (62.0%)	63 (20–87)	*n*/A	267 (75.6%)	93 (26.4%)
	or 341 *	210 (86.1%)		*n*/A	255 (74.8%)	92 (27%)
Saarland	58	33 (57–58%)	64 (27–87)	*n*/A	26 (45.6%)	15 (26%)
	or 57 *			*n*/A	25 (43.9%)	14 (25%)
Heidelberg	59	38 (64.4%)	66 (30–84)	W 59 (100%)	34 (57.6%)	11 (18.6%)
Total	889 or 789 *	522 (58.7%) or 468 (59.3%)	61.4 (18–90) ^#^	W 313 (65.5%) ^$^ B 115 (24.1%) H 22 (4.6%) A 21 (4.4%) O 7 (1.5%)	503 (56.6%) or 403 (51.1%)	163 (18.3%) or 147 (18.6%)

* When stratifying based on the result of blood cultures, 100 sepsis patients without reported blood culture results were excluded. ^#^ The weighted median was used as a point estimate of the median age of the entire study group. ^$^ The race/ethnicity stratification used all the available data from the entire cohort.

**Table 6:** Comparative LR+ ranges for blood-based immune response sepsis biomarkers. Data are summarized from the present paper (SeptiCyte RAPID), from the FDA Decision Summary (Cytovale Intellisep), or the scientific literature (others).

Blood Biomarker	Best Reported LR+	Reference
SeptiCyte RAPID	LR+ ~ 4.4–15.6 (Band 4)	This paper (Figure 2)
pro-BNP	LR+ 1.52 (at cut-off: 2800 pg/mL)	Zhang et al. (2019) [23]
Neutrophil-to-lymphocyte ratio (NLR)	LR+ 1.18 (at cut-off 3) LR+ 2.24 (at cut-off 10)	Balakrishnan et al. (2024) [24]
CRP	LR+ 1.03 (at cut-off: 20 mg/L) LR+ 3.75	Ljungstrom et al. (2017) [25] Ahuja et al. (2023) [26]
Procalcitonin (PCT)	LR+ ~2–3 (10 to >20 ng/mL PCT threshold)	Karlsson et al. (2010) [27]
Cytovale Intellisep	LR+ ~2.7 (Band 3)	K220991 (2022) [28]
IL-6	LR+ 2.36 (1.16–4.80) (pooled studies/meta-analyses)	Hou et al. (2015) [29]
Presepsin (sCD14-ST)	LR+ 3.9	Zhang et al. (2015) [30]
Pancreatic stone protein	LR+ 4.1 (pooled meta-analysis)	Mai et al. (2024) [31]
CD-64	LR+ 8.15 (pooled studies/meta-analyses)	Wang et al. (2015) [32]

**Table 7:** Comparative LR− ranges for blood-based immune response sepsis biomarkers. Data are summarized from the present paper (SeptiCyte RAPID), from FDA Decision Summary (Cytovale Intellisep), or the scientific literature (others).

Blood Host-Response Biomarker	Best Reported LR−	Reference
SeptiCyte RAPID	Sepsis vs. SIRS Band 1: LR− 0.08, Band 2: LR− 0.16 (median values) BC+ (sepsis) vs. SIRS Band 1: LR− 0.02, Band 2: LR− 0.04 (median values) BC− (sepsis) vs. SIRS Band 1: LR− 0.11, Band 2: LR− 0.18 (median values)	This paper (Figure 4 and Figure 5)
Procalcitonin (PCT)	LR− 0.83 (at cut-off: 2 ng/mL)	Ljungstrom et al. (2017) [25]
CRP	LR− 0.82 (at cut-off: 20 mg/L) LR− 0.31	Ljungstrom et al. (2017) [25] Ahuja et al. (2023) [26]
pro-BNP	LR− 0.51 (at cut-off: 2800 pg/mL)	Zhang et al. (2019) [23]
Cytovale Intellisep	LR− 0.35 (Band 1)	K220991 (2022) [28]
IL-6	LR− 0.33 (0.23–0.47) (pooled studies/meta-analyses)	Hou et al. (2015) [29]
Presepsin (sCD14-ST)	LR− 0.27	Zhang et al. (2015) [30]
Pancreatic stone protein	LR− 0.16–0.19 (pooled meta-analysis)	Mai et al. (2024) [31]
Neutrophil-to-lymphocyte ratio (NLR)	LR− 0.17 (at cut-off: 3)	Balakrishnan et al. (2024) [24]
Neutrophil CD-64 (nCD64)	LR− 0.16 (pooled studies/meta-analyses)	Wang et al. (2015) [32]

## Data Availability

The datasets presented in this article are not readily available for patient privacy and commercial reasons. Requests to access the datasets should be directed to Richard Brandon.

## Supplementary Materials

The following supporting information can be downloaded at: https://www.mdpi.com/article/10.3390/diagnostics16152450/s1, Section S1: Definition of SeptiScore Band boundaries. Figure S1: Plot of log(LR+) as a function of SeptiScore. Table S2: Numbers of SIRS & sepsis patients per SeptiScore interval, in the LR analysis of sepsis vs. SIRS. Table S3: Numbers of SIRS & sepsis patients per SeptiScore interval, in the LR analysis of BC (+) sepsis vs. SIRS. Table S4: Numbers of SIRS & sepsis patients per SeptiScore interval, in the LR analysis of BC (−) sepsis vs. SIRS. Section S5: Further analysis of J-shaped curve in Figure 5C. Section S6: Comparative estimates of sepsis pre-test probability. Table S5: Factors reported to affect the estimates of sepsis incidence (pre-test probability). Section S7: Cohort-level heterogeneity analysis of LR+ and LR− across the four component cohorts. Figure S7A: Forest plot of cohort-specific LR− at the Band1/2 boundary (SeptiScore = 5.0; rule-out threshold). Figure S7B: Forest plot of cohort-specific LR+ at the Band3/4 boundary (SeptiScore = 7.4; rule-in threshold). Figure S7C: Combined Forest plots of cohort-specific LR+ and LR− at all three pre-specified SeptiScore Band-boundary cutoffs (5.0, 6.2, 7.4). Table S7a. LR+ estimates [95% CI] by cohort and SeptiScore cutoff, with pooled random-effects estimate and heterogeneity statistics. Table S7b. LR− estimates [95% CI] by cohort and SeptiScore cutoff, with pooled random-effects estimate and heterogeneity statistics. Section S8: Antibiotic timing analysis by cohort [38,39,40,41,42,43].
