# Supplementary material for: Ruling In and Ruling Out Sepsis Using Likelihood Ratios of a Host Response Assay
Source: Diagnostics (Basel). 2026 Aug 3;16(15):2450. doi: 10.3390/diagnostics16152450 (PMC13465465; doi:10.3390/diagnostics16152450)
Supplement: Supplementary file 1 [file diagnostics-16-02450-s001.zip › diagnostics-4423218-supplementary.pdf]

Ruling In and Ruling Out Sepsis Using Likelihood Ratios of a Host Response Assay

SUPPLEMENTARY MATERIAL

Supplement #	Topic
S1	Description of SeptiScore Band Boundaries
S2	Numbers of SIRS & sepsis patients per SeptiScore interval, in the LR analysis of sepsis vs. SIRS
S3	Numbers of SIRS & sepsis patients per SeptiScore interval, in the LR analysis of BC(+) sepsis vs. SIRS
S4	Numbers of SIRS & sepsis patients per SeptiScore interval, in the LR analysis of BC(-) sepsis vs. SIRS
S5	Further analysis of J-shaped Curve in Figure 5C
S6	Comparative estimates of pre-test probability of sepsis
S7	Cohort-Level Heterogeneity Analysis of LR+ and LR−
S8	Antibiotic-Timing Analysis by Cohort

Section S1: Description of SeptiScore Band Boundaries

Much of the analysis and discussion in this paper centers around the concept of "SeptiScore Bands". Four SeptiScore Bands and their boundaries had been pre-defined, in the following way, as described previously in Balk et al. (2024) [1]. An independent set of 195 patient samples that had been adjudicated by RPD was used to set the Band 1/2 cutoff at 5.0 to achieve 90% sensitivity, and the Band 3/4 cutoff at 7.4 to achieve 80% specificity. The Band 2/3 cutoff was then defined as the midpoint between these two outer thresholds.

For descriptive comparison, Figure S1 shows the relationship between LR+ and SeptiScore on a semilogarithmic scale. This visualization indicates that the previously defined Band 1/2 and Band 3/4 boundaries fall in regions where the slope of the log(LR+) versus SeptiScore curve appears to change. This observation is presented as a qualitative consistency check between the pre-specified band structure and the behavior of LR+ across the SeptiScore range.

Figure S1: Plot of log(LR+) as a function of SeptiScore

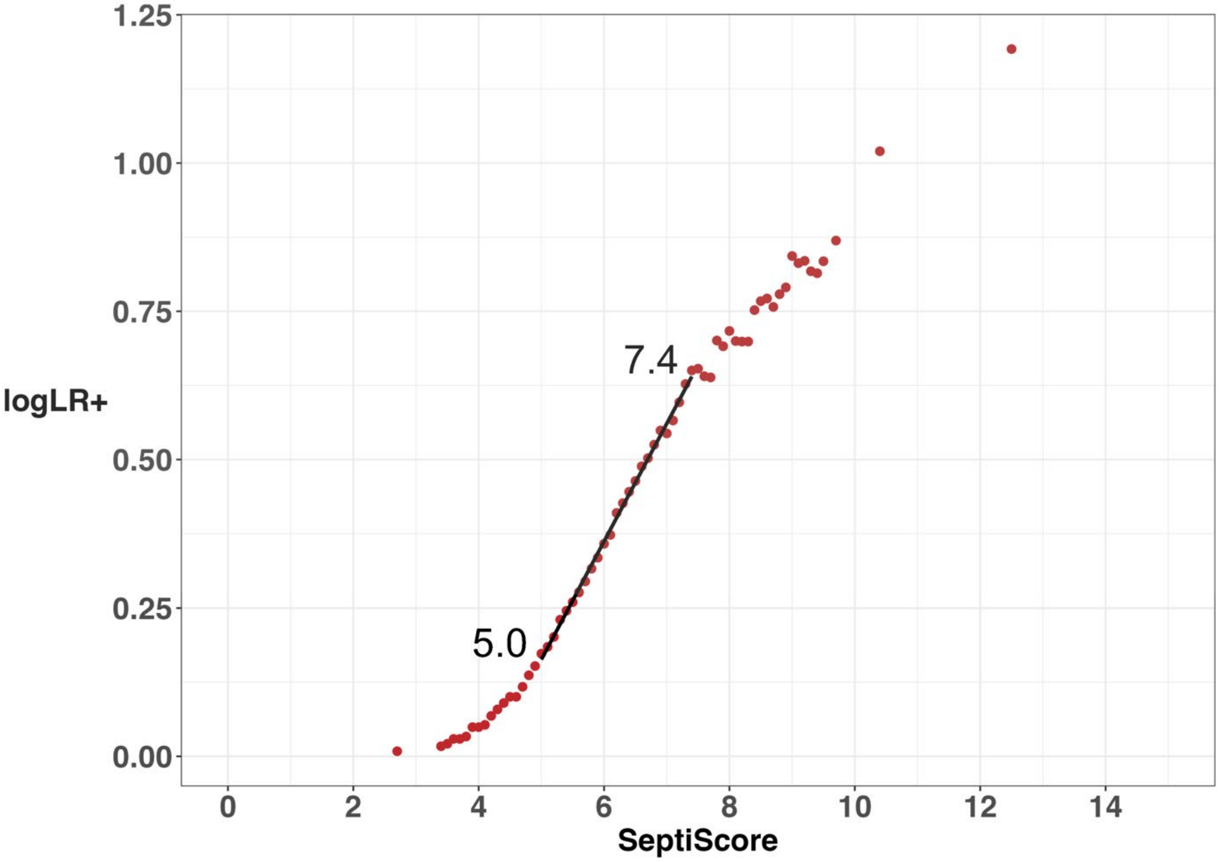

Table S2: Numbers of sepsis & SIRS patients per SeptiScore interval, in the LR analysis of sepsis vs. SIRS. The dataset for this analysis consists of N=889 patients. Likelihood ratio estimates at the extreme ends of the SeptiScore distribution are based on wide SeptiScore intervals to allow sufficient numbers of SIRS & sepsis patients per interval for accurate LR estimation.

SeptiScore range (midpoint)	RAPID Interpretation Band	Likelihood ratio (positive)	Likelihood ratio (negative)	No. of Sepsis patients	No. of SIRS patients	Total No. patients
1.8-3.3 (mid 2.7)	Band 1	1.02	0.014	2	15	17
3.4	Band 1	1.04	0.102	0	5	5
3.5	Band 1	1.05	0.077	1	6	7
3.6	Band 1	1.07	0.089	0	3	3
3.7	Band 1	1.07	0.079	1	4	5
3.8	Band 1	1.08	0.093	0	11	11
3.9	Band 1	1.12	0.070	1	1	2
4	Band 1	1.12	0.085	3	4	7
4.1	Band 1	1.13	0.125	3	14	17

SeptiScore range (midpoint)	RAPID Interpretation Band	Likelihood ratio (positive)	Likelihood ratio (negative)	No. of Sepsis patients	No. of SIRS patients	Total No. patients
4.2	Band 1	1.17	0.134	1	10	11
4.3	Band 1	1.2	0.126	0	7	7
4.4	Band 1	1.23	0.115	3	8	11
4.5	Band 1	1.26	0.131	4	4	8
4.6	Band 1	1.26	0.158	2	12	14
4.7	Band 1	1.31	0.155	2	13	15
4.8	Band 1	1.37	0.151	0	10	10
4.9	Band 1	1.42	0.139	2	12	14
5	Band 2	1.49	0.138	3	8	11
5.1	Band 2	1.53	0.146	1	10	11
5.2	Band 2	1.59	0.142	2	16	18
5.3	Band 2	1.7	0.138	1	8	9
5.4	Band 2	1.76	0.136	5	9	14
5.5	Band 2	1.82	0.149	6	9	15
5.6	Band 2	1.89	0.166	3	9	12
5.7	Band 2	1.97	0.170	3	10	13
5.8	Band 2	2.07	0.172	6	9	15
5.9	Band 2	2.16	0.186	5	10	15
6	Band 2	2.28	0.194	4	6	10
6.1	Band 2	2.36	0.202	3	13	16
6.2	Band 3	2.57	0.201	4	6	10
6.3	Band 3	2.67	0.208	6	7	13
6.4	Band 3	2.79	0.220	2	5	7
6.5	Band 3	2.91	0.221	7	8	15
6.6	Band 3	3.08	0.234	7	5	12
6.7	Band 3	3.18	0.249	4	6	10
6.8	Band 3	3.35	0.254	9	7	16
6.9	Band 3	3.54	0.271	14	2	16
7	Band 3	3.5	0.305	9	6	15
7.1	Band 3	3.68	0.321	9	7	16
7.2	Band 3	3.95	0.336	6	6	12
7.3	Band 3	4.24	0.344	4	4	8
7.4	Band 4	4.47	0.349	9	2	11
7.5	Band 4	4.5	0.368	16	1	17
7.6	Band 4	4.37	0.405	7	1	8
7.7	Band 4	4.35	0.420	9	9	18

SeptiScore range (midpoint)	RAPID Interpretation Band	Likelihood ratio (positive)	Likelihood ratio (negative)	No. of Sepsis patients	No. of SIRS patients	Total No. patients
7.8	Band 4	5.02	0.429	7	0	7
7.9	Band 4	4.91	0.445	8	4	12
8	Band 4	5.21	0.458	12	0	12
8.1	Band 4	5.01	0.485	7	1	8
8.2	Band 4	5	0.499	13	2	15
8.3	Band 4	5	0.525	2	5	7
8.4	Band 4	5.65	0.522	6	2	8
8.5	Band 4	5.85	0.532	5	1	6
8.6	Band 4	5.91	0.541	8	0	8
8.7	Band 4	5.72	0.559	11	3	14
8.8	Band 4	6.01	0.578	10	2	12
8.9	Band 4	6.17	0.596	7	4	11
9	Band 4	6.97	0.604	6	0	6
9.1	Band 4	6.78	0.617	7	1	8
9.2	Band 4	6.84	0.630	8	0	8
9.3	Band 4	6.57	0.647	10	1	11
9.4	Band 4	6.52	0.666	18	3	21
9.5	Band 4	6.83	0.698	8	1	9
9.6-9.9 (mid 9.7)	Band 4	7.4	0.723	21	6	27
10.0-10.9 (mid 10.4)	Band 4	10.47	0.795	60	6	66
11.0-15.0 (mid 12.5)	Band 4	15.57	0.944	80	6	86
			**Total**	**503**	**386**	**889**

Table S3: Numbers of SIRS & sepsis patients per SeptiScore interval, in the LR analysis of BC(+) sepsis vs. SIRS. The dataset for this analysis consists of N=570 patients. Likelihood ratio estimates at the extreme ends of the SeptiScore distribution are based on wide SeptiScore intervals to allow sufficient numbers of SIRS & sepsis patients per interval for accurate LR estimation.

SeptiScore range (midpoint)	RAPID Interpretation Band	Likelihood ratio (positive)	Likelihood ratio (negative)	No. of BC+ Sepsis patients	No. of SIRS patients	Total No. patients
1.8-3.3 (mid 2.7)	Band 1	1.02	0.000	1	15	16

SeptiScore range (midpoint)	RAPID Interpretation Band	Likelihood ratio (positive)	Likelihood ratio (negative)	No. of BC+ Sepsis patients	No. of SIRS patients	Total No. patients
3.4	Band 1	1.03	0.140	0	5	5
3.5	Band 1	1.05	0.105	0	6	6
3.6	Band 1	1.07	0.081	0	3	3
3.7	Band 1	1.08	0.072	0	4	4
3.8	Band 1	1.09	0.064	0	11	11
3.9	Band 1	1.12	0.048	0	1	1
4	Band 1	1.13	0.047	0	4	4
4.1	Band 1	1.14	0.043	0	14	14
4.2	Band 1	1.19	0.033	0	10	10
4.3	Band 1	1.23	0.029	0	7	7
4.4	Band 1	1.25	0.026	0	8	8
4.5	Band 1	1.29	0.024	0	4	4
4.6	Band 1	1.31	0.023	0	12	12
4.7	Band 1	1.36	0.020	0	13	13
4.8	Band 1	1.43	0.018	0	10	10
4.9	Band 1	1.48	0.017	0	12	12
5	Band 2	1.55	0.015	1	8	9
5.1	Band 2	1.6	0.029	0	10	10
5.2	Band 2	1.67	0.027	0	16	16
5.3	Band 2	1.79	0.024	0	8	8
5.4	Band 2	1.86	0.023	1	9	10
5.5	Band 2	1.94	0.033	2	9	11
5.6	Band 2	2.01	0.053	1	9	10
5.7	Band 2	2.1	0.061	1	10	11
5.8	Band 2	2.21	0.067	4	9	13
5.9	Band 2	2.28	0.102	2	10	12
6	Band 2	2.41	0.115	0	6	6
6.1	Band 2	2.51	0.112	0	13	13
6.2	Band 3	2.76	0.107	3	6	9
6.3	Band 3	2.84	0.128	2	7	9
6.4	Band 3	2.98	0.140	0	5	5
6.5	Band 3	3.11	0.138	2	8	10
6.6	Band 3	3.31	0.149	2	5	7
6.7	Band 3	3.43	0.161	2	6	8
6.8	Band 3	3.61	0.172	2	7	9
6.9	Band 3	3.85	0.182	3	2	5

SeptiScore range (midpoint)	RAPID Interpretation Band	Likelihood ratio (positive)	Likelihood ratio (negative)	No. of BC+ Sepsis patients	No. of SIRS patients	Total No. patients
7	Band 3	3.87	0.201	4	6	10
7.1	Band 3	4.06	0.225	3	7	10
7.2	Band 3	4.37	0.240	3	6	9
7.3	Band 3	4.68	0.255	0	4	4
7.4	Band 4	4.99	0.252	3	2	5
7.5	Band 4	5.05	0.269	8	1	9
7.6	Band 4	4.85	0.320	1	1	2
7.7	Band 4	4.89	0.325	4	9	13
7.8	Band 4	5.64	0.341	2	0	2
7.9	Band 4	5.55	0.354	4	4	8
8	Band 4	5.86	0.374	5	0	5
8.1	Band 4	5.63	0.405	1	1	2
8.2	Band 4	5.71	0.410	9	2	11
8.3	Band 4	5.53	0.462	0	5	5
8.4	Band 4	6.29	0.456	1	2	3
8.5	Band 4	6.6	0.459	2	1	3
8.6	Band 4	6.67	0.469	3	0	3
8.7	Band 4	6.48	0.487	2	3	5
8.8	Band 4	6.99	0.495	5	2	7
8.9	Band 4	7.12	0.522	3	4	7
9	Band 4	8.04	0.533	2	0	2
9.1	Band 4	7.87	0.545	0	1	1
9.2	Band 4	8.21	0.543	4	0	4
9.3	Band 4	7.84	0.566	4	1	5
9.4	Band 4	7.82	0.588	10	3	13
9.5	Band 4	7.95	0.640	5	1	6
9.6-9.9 (mid 9.7)	Band 4	8.4	0.678	10	6	16
10.0-10.9 (mid 10.4)	Band 4	11.18	0.779	27	6	33
11.0-15.0 (mid 12.5)	Band 4	20.16	0.929	30	6	36
				**184**	**386**	**570**

Table S4: Numbers of SIRS & sepsis patients per SeptiScore interval, in the LR analysis of BC(-) sepsis vs. SIRS. The dataset for this analysis consists of N=605 patients. Likelihood ratio estimates at the extreme ends of the SeptiScore distribution are based on wide SeptiScore intervals to allow sufficient numbers of SIRS & sepsis patients per interval for accurate LR estimation.

SeptiScore range (midpoint)	RAPID Interpretation Band	Likelihood ratio (positive)	Likelihood ratio (negative)	No. of BC- Sepsis patients	No. of SIRS patients	Total No. patients
1.8-3.3 (mid 2.7)	Band 1	1.02	0.031	1	15	16
3.4	Band 1	1.04	0.118	0	5	5
3.5	Band 1	1.05	0.088	0	6	6
3.6	Band 1	1.07	0.068	0	3	3
3.7	Band 1	1.08	0.061	1	4	5
3.8	Band 1	1.08	0.107	0	11	11
3.9	Band 1	1.12	0.080	1	1	2
4	Band 1	1.12	0.118	1	4	5
4.1	Band 1	1.12	0.144	2	14	16
4.2	Band 1	1.16	0.168	0	10	10
4.3	Band 1	1.2	0.145	0	7	7
4.4	Band 1	1.23	0.132	1	8	9
4.5	Band 1	1.25	0.140	2	4	6
4.6	Band 1	1.26	0.172	2	12	14
4.7	Band 1	1.3	0.186	0	13	13
4.8	Band 1	1.36	0.166	0	10	10
4.9	Band 1	1.42	0.153	2	12	14
5	Band 2	1.47	0.165	0	8	8
5.1	Band 2	1.52	0.156	1	10	11
5.2	Band 2	1.58	0.157	1	16	17
5.3	Band 2	1.69	0.153	1	8	9
5.4	Band 2	1.75	0.156	1	9	10
5.5	Band 2	1.82	0.158	2	9	11
5.6	Band 2	1.89	0.168	1	9	10
5.7	Band 2	1.97	0.169	1	10	11
5.8	Band 2	2.08	0.170	1	9	10
5.9	Band 2	2.18	0.171	3	10	13
6	Band 2	2.29	0.186	4	6	10

SeptiScore range (midpoint)	RAPID Interpretation Band	Likelihood ratio (positive)	Likelihood ratio (negative)	No. of BC-Sepsis patients	No. of SIRS patients	Total No. patients
6.1	Band 2	2.34	0.210	2	13	15
6.2	Band 3	2.55	0.213	0	6	6
6.3	Band 3	2.67	0.209	3	7	10
6.4	Band 3	2.79	0.223	1	5	6
6.5	Band 3	2.9	0.225	5	8	13
6.6	Band 3	3.03	0.250	2	5	7
6.7	Band 3	3.15	0.258	1	6	7
6.8	Band 3	3.34	0.259	2	7	9
6.9	Band 3	3.57	0.264	7	2	9
7	Band 3	3.5	0.303	4	6	10
7.1	Band 3	3.68	0.320	4	7	11
7.2	Band 3	3.95	0.336	0	6	6
7.3	Band 3	4.31	0.329	4	4	8
7.4	Band 4	4.48	0.347	4	2	6
7.5	Band 4	4.51	0.367	5	1	6
7.6	Band 4	4.44	0.392	4	1	5
7.7	Band 4	4.39	0.413	5	9	14
7.8	Band 4	5.03	0.428	5	0	5
7.9	Band 4	4.85	0.454	2	4	6
8	Band 4	5.21	0.459	4	0	4
8.1	Band 4	5.05	0.479	4	1	5
8.2	Band 4	5	0.498	2	2	4
8.3	Band 4	5.16	0.506	2	5	7
8.4	Band 4	5.78	0.509	4	2	6
8.5	Band 4	5.91	0.526	2	1	3
8.6	Band 4	5.98	0.534	4	0	4
8.7	Band 4	5.77	0.554	6	3	9
8.8	Band 4	5.99	0.579	4	2	6
8.9	Band 4	6.17	0.596	4	4	8
9	Band 4	6.9	0.609	2	0	2
9.1	Band 4	6.76	0.618	4	1	5
9.2	Band 4	6.74	0.636	3	0	3
9.3	Band 4	6.51	0.651	5	1	6

SeptiScore range (midpoint)	RAPID Interpretation Band	Likelihood ratio (positive)	Likelihood ratio (negative)	No. of BC-Sepsis patients	No. of SIRS patients	Total No. patients
9.4	Band 4	6.41	0.673	7	3	10
9.5	Band 4	6.77	0.701	2	1	3
9.6-9.9 (mid 9.7)	Band 4	7.66	0.718	10	6	16
10.0-10.9 (mid 10.4)	Band 4	10.85	0.784	24	6	30
11.0-15.0 (mid 12.5)	Band 4	14.67	0.935	37	6	43
				**219**	**386**	**605**

Section S5: Further analysis of J-shaped Curve in Figure 5C

In Figure 5C, the LR^- analysis for the BC(+) versus SIRS comparison, we observe a distinctive J-shaped curve with a minimum near 5.0 (Figure 5C, blue points). To understand this behavior, we examined the components of the LR^- formula, LR- = (FN/TN) x (TN+FP) / (TP+FN). The second term is constant, because TN+FP equals the total number of negative calls. The third term is also constant, because TP+FN equals the total number of positive calls. Consequently, all variation in LR^- must arise from the FN/TN term. When FN/TN is plotted as a function of SeptiScore, its curve closely mirrors the LR^- curve (Figure 5C, orange points, reproduced below).

The "J" shape of the curve between the limits of 5.0 and 3.3 easily explained, as follows. The second-lowest BC(+) sepsis patient has SeptiScore = 5.0, and the lowest BC(+) sepsis patient has SeptiScore 3.3. As the cutoff is decreased below 5.0 toward 3.3, the numerator of FN/TN remains constant at FN=1 (because there is only the single BC(+) sepsis patient remaining, while the denominator TN (equal to the number of true negatives below the cutoff) continues to decrease. Thus, the ratio FN/TN increases monotonically as the cutoff is lowered.

Figure 5C in the main text:

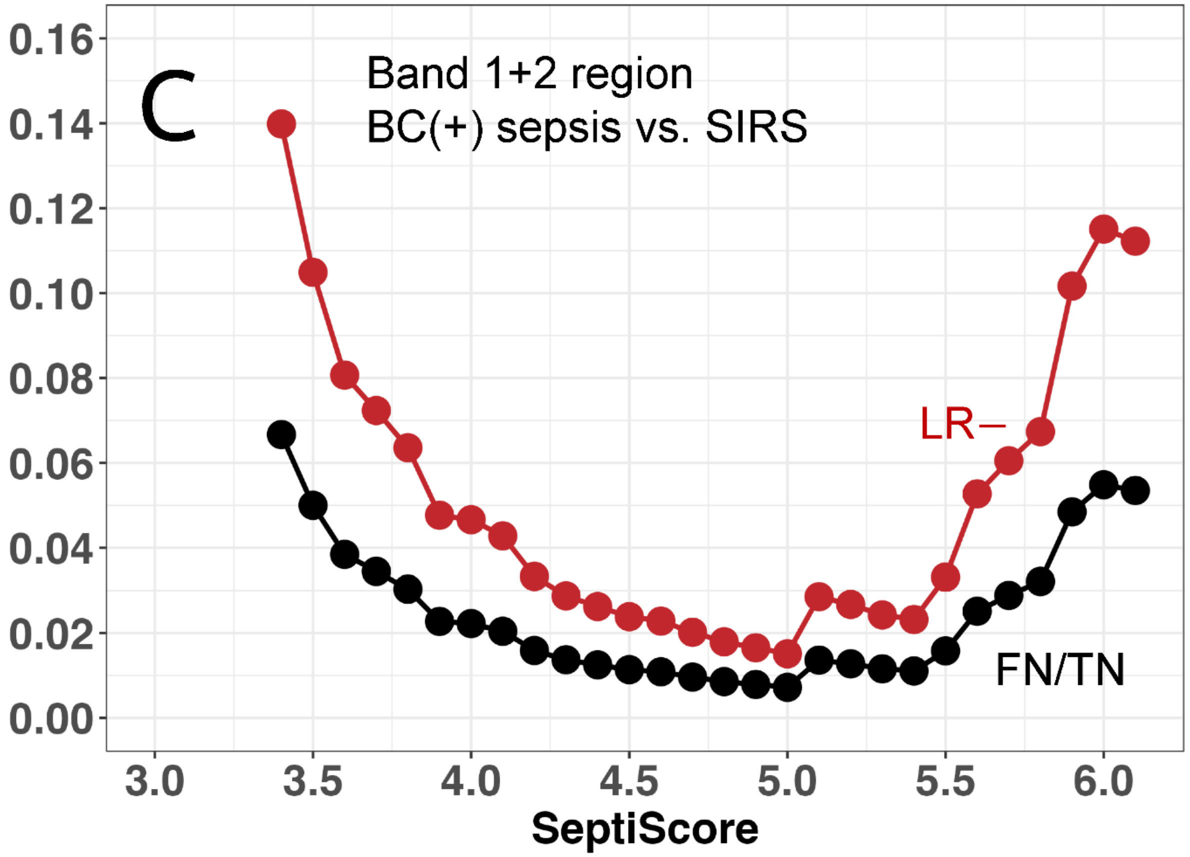

Section S6: Comparative Estimates of Sepsis pre-test Probability

In the acute care setting considered here, the relevant time horizon is very short—approximately ±1 day around the attending physician's decision to initiate or withhold a sepsis bundle (including intravenous antibiotics and microbiological cultures) in response to a suspected new sepsis event. Over such a narrow interval, the pre-test probability can be reasonably approximated by the short-interval incidence of sepsis, as disease status evolves rapidly and the distinction between point prevalence and incidence becomes negligible at this time scale. Accordingly, for the purposes of the present analysis, we assume the pre-test probability to be the cohort-level short-interval incidence of sepsis, as determined by expert adjudication.

We wish to place the estimated pre-test probability of sepsis (50% ± 2.5%) into the broader context of what might be expected on the basis of other studies from the literature. In the Intensive Care over Nations (ICON) audit (Sakr et al., 2018) [33], estimates of sepsis incidence in the ICU vary widely, with an average incidence of 29.5% and a range of 13.6% to 39.3% depending upon region. This variation most likely depends on multiple factors, as enumerated in the following table.

Table S5: Factors reported to affect the estimates of sepsis incidence (pre-test probability)

**Influencing Factor**	**Likely effect**	**Reference**
Framework definition: sepsis-2 vs. sepsis-3	Sepsis-3 more restrictive, leading to fewer patients called septic, therefore lower incidence rate	Shankar-Hari et al. (2017) [39]<br>Hammond et al. (2022) [40]

Influencing Factor	Likely effect	Reference
ICD code-based vs. chart-based review	Chart review more accurate, but more resource intensive.	Mariansdatter et al. (2016) [41]
Cohort specific factor: comorbidities	Sepsis incidence increases with particular comorbidities: COPD, heart failure, renal failure, cirrhosis, immunosuppression, HIV-1 infection	Sakr et al. (2018) [33]
Cohort specific factor: geographic location	Higher infectious disease burden in some locations leads to higher sepsis incidence	Sakr et al. (2018) [33]<br>GBD (2021) [42]
Cohort specific factor: race/ethnicity	Higher incidence of severe sepsis reported for Black, as opposed to Hispanic or White (root cause not identified)	Barnato et al. (2008) [43]

Section S7: Cohort-Level Heterogeneity Analysis of LR+ and LR−

In the main text, we have pooled data from four independent patient cohorts (510(k), Andalusia, Heidelberg, Saarland) to estimate SeptiCyte RAPID's likelihood ratios (LRs). Because the cohorts differ in geography, era, and sepsis-adjudication method, a natural question is whether they actually agree with each other, or whether pooling has masked important disagreement between sites. This Supplement answers that question two ways. (1) We recomputed the LR at each of the three main SeptiScore Band-boundary cutoffs separately within each cohort and compare the four cohort-specific values using forest plots and two formal heterogeneity statistics, Cochran's Q and I^2. (2) Additionally, as a threshold-free check, we test statistically whether the overall relationship between SeptiScore and sepsis status differs by cohort at all. Both approaches point to the same conclusion: cohort-to-cohort agreement is strong at the rule-out threshold and reasonably consistent overall, supporting the validity of pooling these cohorts in the main analysis.

A "Forest plot" is a standard way, widely used in meta-analysis, for displaying several studies' (here, cohorts') effect estimates side-by-side, along with a combined estimate. In each plot, the blue squares mark each cohort's own LR point estimate, with the square's size scaled to that cohort's weight in the pooled analysis (larger cohorts / more precise estimates get bigger squares); the horizontal line through each square (the "whiskers") spans that cohort's 95% confidence interval; the red diamond marks the pooled random-effects estimate, with the diamond's width spanning its own 95% CI; and bracketed values [ ] throughout this Supplement denote 95% confidence intervals.

Agreement across cohorts was quantified using Cochran's Q (a formal test of whether the cohort-specific estimates differ from each other more than would be expected by chance alone) and I^2 (the percentage of the total variability across cohorts that reflects genuine between-cohort differences rather than chance; $I^2 = 100 \times (Q - df)/Q$, where df is Q's degrees of freedom, i.e. the number of cohorts minus one).

In the analysis presented in the main text, data from four independent cohorts were pooled. In this Supplement, we demonstrate that likelihood ratio (LR) estimates calculated separately from the individual cohort data are consistent with each other. Assessment was made at each of the three pre-specified SeptiScore Band-boundary cutoffs (SeptiScore 5.0 = Band1/2 cutoff; Septicore 6.2 = Band2/3 cutoff; SeptiScore 7.4 = Band3/4 cutoff). At At each cutoff, cohort-specific LR+ and LR− were computed from cohort-specific 2×2 contingency tables, with log-scale standard errors derived following Simel et al.

(1991) and Deeks and Altman (2004) [3]. A 0.5 continuity correction was applied only where a contingency cell was zero. Cohort-specific estimates were then combined into a single pooled random-effects estimate -- a weighted average across the four cohorts, computed using the DerSimonian and Laird (1986) [13] method, that explicitly allows for and accounts for between-cohort variability rather than assuming all four cohorts share one identical true LR. As a complement to this threshold-based analysis, we also asked a broader question: does the overall relationship between SeptiScore and sepsis status differ by cohort at all, beyond these three specific cutoffs? We tested this with a logistic regression model that included a SeptiScore-by-cohort interaction term, comparing it via likelihood-ratio test to the corresponding model without that interaction term; a statistically significant result would indicate that the SeptiScore-sepsis relationship itself differs by cohort.

At the Band1/2 boundary (SeptiScore = 5.0), which underlies the rule-out claim, cohort-specific LR− estimates were highly consistent ($I^2 = 5\%$, Cochran's Q = 3.16, df = 3, p = 0.367; pooled LR− = 0.16, 95% CI 0.11–0.25). At the Band3/4 boundary (SeptiScore = 7.4), which underlies the rule-in claim, cohort-specific LR+ estimates showed moderate, borderline heterogeneity ($I^2 = 53\%$, Cochran's Q = 6.39, df = 3, p = 0.094; pooled LR+ = 3.57, 95% CI 2.49–5.14), driven mainly by somewhat higher point estimates in the two larger cohorts (510k, Andalusia) than in the two smaller cohorts (Heidelberg, Saarland). The intermediate Band2/3 boundary (6.2) showed a similar pattern for LR+ ($I^2 = 53\%$, p = 0.093) and greater heterogeneity for LR− ($I^2 = 79\%$, p = 0.003), as did LR− at the Band3/4 boundary ($I^2 = 82\%$, p = 0.001). The threshold-free interaction test described above was not statistically significant (likelihood-ratio $\chi^2 = 5.27$, df = 3, p = 0.153) -- that is, we found no evidence that the overall SeptiScore-sepsis relationship differs systematically by cohort, beyond the boundary-specific heterogeneity described above.

Figure S7A: Forest plot of cohort-specific LR− at the Band1/2 boundary (SeptiScore = 5.0; rule-out threshold).

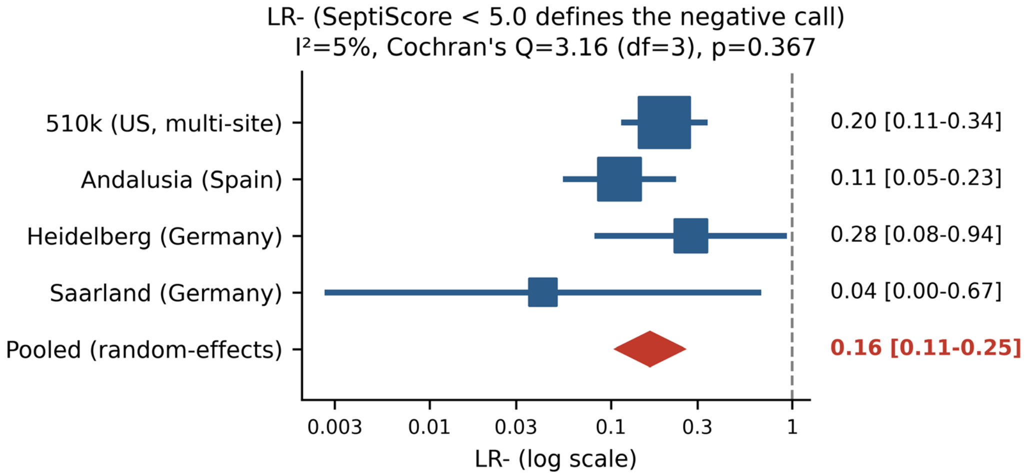

Figure S7B: Forest plot of cohort-specific LR+ at the Band3/4 boundary (SeptiScore = 7.4; rule-in threshold).

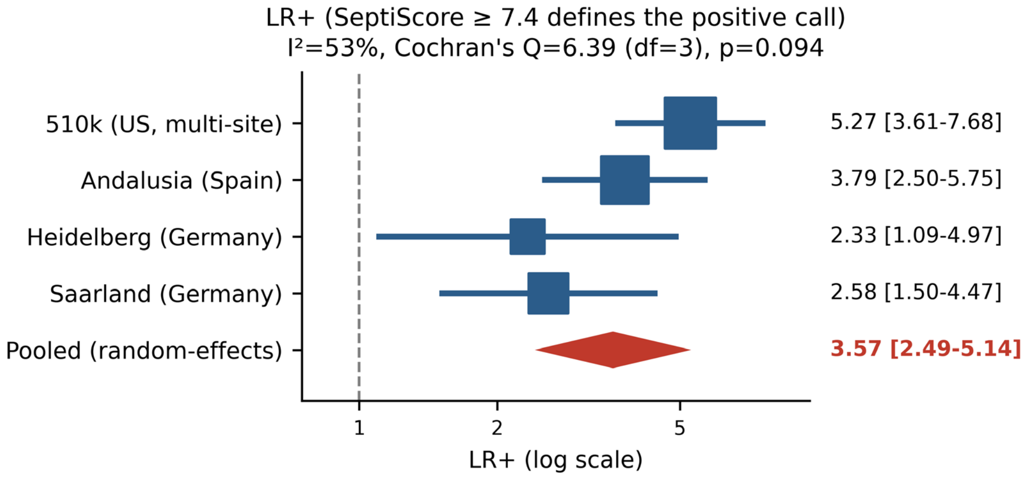

Figure S7C: Combined Forest plots of cohort-specific LR+ and LR− at all three pre-specified SeptiScore Band-boundary cutoffs (5.0, 6.2, 7.4).

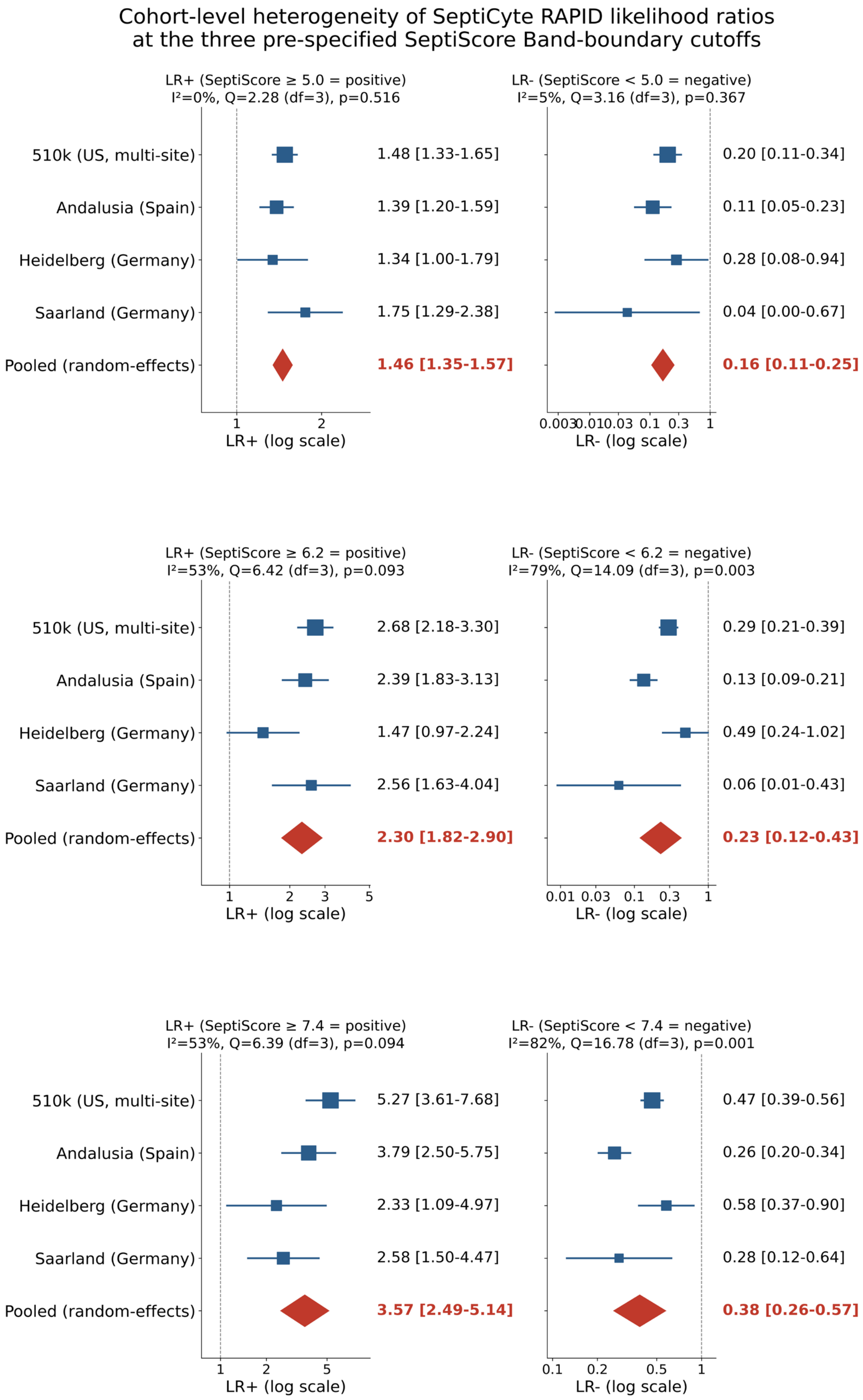
Cohort-level heterogeneity of SeptiCyte RAPID likelihood ratios
at the three pre-specified SeptiScore Band-boundary cutoffs
LR+ (SeptiScore ≥ 5.0 = positive)
I²=0%, Q=2.28 (df=3), p=0.516
LR- (SeptiScore < 5.0 = negative)
I²=5%, Q=3.16 (df=3), p=0.367
510k (US, multi-site)
Andalusia (Spain)
Heidelberg (Germany)
Saarland (Germany)
Pooled (random-effects)
1.48 [1.33-1.65]
1.39 [1.20-1.59]
1.34 [1.00-1.79]
1.75 [1.29-2.38]
1.46 [1.35-1.57]
0.20 [0.11-0.34]
0.11 [0.05-0.23]
0.28 [0.08-0.94]
0.04 [0.00-0.67]
0.16 [0.11-0.25]
1
2
LR+ (log scale)
0.003
0.01
0.03
0.1
0.3
1
LR- (log scale)
LR+ (SeptiScore ≥ 6.2 = positive)
I²=53%, Q=6.42 (df=3), p=0.093
LR- (SeptiScore < 6.2 = negative)
I²=79%, Q=14.09 (df=3), p=0.003
510k (US, multi-site)
Andalusia (Spain)
Heidelberg (Germany)
Saarland (Germany)
Pooled (random-effects)
2.68 [2.18-3.30]
2.39 [1.83-3.13]
1.47 [0.97-2.24]
2.56 [1.63-4.04]
2.30 [1.82-2.90]
0.29 [0.21-0.39]
0.13 [0.09-0.21]
0.49 [0.24-1.02]
0.06 [0.01-0.43]
0.23 [0.12-0.43]
1
2
3
5
LR+ (log scale)
0.01
0.03
0.1
0.3
1
LR- (log scale)
LR+ (SeptiScore ≥ 7.4 = positive)
I²=53%, Q=6.39 (df=3), p=0.094
LR- (SeptiScore < 7.4 = negative)
I²=82%, Q=16.78 (df=3), p=0.001
510k (US, multi-site)
Andalusia (Spain)
Heidelberg (Germany)
Saarland (Germany)
Pooled (random-effects)
5.27 [3.61-7.68]
3.79 [2.50-5.75]
2.33 [1.09-4.97]
2.58 [1.50-4.47]
3.57 [2.49-5.14]
0.47 [0.39-0.56]
0.26 [0.20-0.34]
0.58 [0.37-0.90]
0.28 [0.12-0.64]
0.38 [0.26-0.57]
1
2
5
LR+ (log scale)
0.1
0.2
0.5
1
LR- (log scale)

Table S7a. LR+ estimates [95% CI] by cohort and SeptiScore cutoff, with pooled random-effects estimate and heterogeneity statistics.

Cutoff	510k (US)	Andalusia (Spain)	Heidelberg (Germany)	Saarland (Germany)	Pooled (random-effects) [95% CI]	I^2 (%)	Cochran's Q (df=3)	Cochran's Q p -value (heterogeneity)
5.0	1.48 [1.33-1.65]	1.39 [1.20-1.59]	1.34 [1.00-1.79]	1.75 [1.29-2.38]	1.46 [1.35-1.57]	0	2.28	0.516
6.2	2.68 [2.18-3.30]	2.39 [1.83-3.13]	1.47 [0.97-2.24]	2.56 [1.63-4.04]	2.30 [1.82-2.90]	53	6.42	0.093
7.4	5.27 [3.61-7.68]	3.79 [2.50-5.75]	2.33 [1.09-4.97]	2.58 [1.50-4.47]	3.57 [2.49-5.14]	53	6.39	0.094

Table S7b. LR− estimates [95% CI] by cohort and SeptiScore cutoff, with pooled random-effects estimate and heterogeneity statistics.

Cutoff	510k (US)	Andalusia (Spain)	Heidelberg (Germany)	Saarland (Germany)	Pooled (random-effects) [95% CI]	I^2 (%)	Cochran's Q (df=3)	Cochran's Q p-value (heterogeneity)
5.0	0.20 [0.11-0.34]	0.11 [0.05-0.23]	0.28 [0.08-0.94]	0.04 [0.00-0.67]	0.16 [0.11-0.25]	5	3.16	0.367
6.2	0.29 [0.21-0.39]	0.13 [0.09-0.21]	0.49 [0.24-1.02]	0.06 [0.01-0.43]	0.23 [0.12-0.43]	79	14.09	0.003
7.4	0.47 [0.39-0.56]	0.26 [0.20-0.34]	0.58 [0.37-0.90]	0.28 [0.12-0.64]	0.38 [0.26-0.57]	82	16.78	0.001

Section S8: Antibiotic-Timing Analysis by Cohort

This section provides the cohort-by-cohort detail supporting the antibiotic-exposure discussion in the main text (Discussion, Limitations). We examined antibiotic-timing data, where available, in three of the four cohorts. In the 510(k) cohort, SeptiScore was drawn after the first antibiotic dose in 77.6% of BC (+) sepsis patients versus 37.5% of BC (−) sepsis patients and 33.7% of SIRS controls, yet SeptiScore did not differ significantly by antibiotic-exposure status within either BC (+) sepsis (median 8.2 vs. 8.1, Mann–Whitney p = 0.49) or BC (−) sepsis (median 7.7 vs. 7.5, p = 0.56). This is consistent with an independent stratified analysis of the same 510(k) cohort (Balk et al., 2024, Figure 4) [38], which found that SeptiCyte RAPID's discriminative performance (AUC 0.79–0.84) was not significantly affected by antibiotic-initiation timing across a window from one day before to one day after blood draw. In the Heidelberg cohort, virtually all BC(+) (15/15) and BC(−) (19/19) sepsis patients had already received antibiotics before sample collection, precluding a within-cohort timing comparison but also ruling out differential antibiotic timing as an explanation for the BC(+)/BC(−) SeptiScore difference observed at that site. The Saarland cohort's enrollment criteria excluded patients with more than 48 hours of prior antimicrobial therapy. Antibiotic-timing data were not available for the Andalusia (Seville) cohort; per the original cohort publication, administration of antibiotics before ICU admission was not recorded, and patients were not excluded on the basis of antibiotic treatment (exclusion criteria were limited to pregnancy and a clinical picture suggestive of sepsis that had started more than 48 h previously). Taken together, these analyses indicate that antibiotic exposure at the time of sampling did not measurably shift SeptiScore within either BC (+) or BC (−) sepsis groups in the cohorts where this could be tested, consistent with SeptiCyte RAPID measuring the host transcriptional response rather than pathogen viability. We recommend that future studies systematically record antibiotic-exposure timing across all cohorts to evaluate this relationship with greater precision.
